# Quantifying the effects of neutron dose, dose protraction, age and sex on mouse survival using parametric regression and machine learning on a 21,000-mouse data set

**DOI:** 10.1038/s41598-023-49262-3

**Published:** 2023-12-09

**Authors:** Eric Wang, Igor Shuryak, David J. Brenner

**Affiliations:** Center for Radiological Research, Columbia University Irving Medical Center, 630 West 168th Street, VC-11, New York, NY 10032 USA

**Keywords:** Computational biology and bioinformatics, Risk factors

## Abstract

The biological effects of densely-ionizing radiations such as neutrons and heavy ions encountered in space travel, nuclear incidents, and cancer radiotherapy, significantly differ from those of sparsely-ionizing photons and necessitate a comprehensive understanding for improved protection measures. Data on lifespan studies of laboratory rodents exposed to fission neutrons, accumulated in the Janus archive, afford unique insights into the impact of densely ionizing radiation on mortality from cancers and various organ dysfunction. We extracted and analyzed data for 21,308 individual B6CF1 mice to investigate the effects of neutron dose, fractionation, protraction, age, and sex on mortality. As Cox regression encountered limitations owing to assumption violations, we turned to Random Survival Forests (RSF), a machine learning algorithm adept at modeling nonlinear relationships. RSF interpretation using Shapley Additive Explanations revealed a dose response for mortality risk that curved upwards at low doses < 20 cGy, became nearly-linear over 20–150 cGy, and saturated at high doses. The response was enhanced by fractionation/protraction of irradiation (exhibiting an inverse dose rate effect), and diminished by older age at exposure. Somewhat reduced mortality was predicted for males vs females. This research expands our knowledge on the long-term effects of densely ionizing radiations on mammal mortality.

## Introduction

Densely ionizing radiations, such as neutrons or heavy ions, can cause not only quantitatively, but potentially qualitatively distinct biological damage, compared to sparsely ionizing photons or electrons^1^. Densely ionizing particles can produce spatially clustered ionizations along defined tracks, while sparsely ionizing radiations tend to create more random distributions of ionizations^2^. These differences in energy deposition between radiation types affect their biological effectiveness. Specifically, damage caused by densely ionizing particles tends to result in more difficult to repair molecular lesions, resulting in more frequent and severe adverse health effects, including carcinogenesis and central nervous system dysfunction, compared to sparsely ionizing radiations^1,2^.

Understanding the effects of densely ionizing radiation is, therefore, crucial for radiation protection in diverse scenarios, including human space exploration missions, nuclear explosions and radioactivity releases on Earth, cancer radiotherapy, and more^2^. In particular, the harmful health effects caused by a mixture of sparsely and densely ionizing radiations present a significant challenge that must be addressed for long-duration space exploration missions, such as a trip to Mars, to be possible^2^.

Data on densely-ionizing radiation effects in humans exist, but are relatively limited, with much of them confined to effects of radon on underground miners and domestic exposures^3–5^ and some medical or accidental exposures to alpha particle emitting radionuclides. For obvious reasons, such human data need to be augmented by experimental irradiation studies in laboratory organisms such as rodents. Specifically, large historical animal data sets such as the neutron irradiation experiments conducted by Argonne National Laboratory offer valuable insights, but there remains a need to adopt innovative methods for analyzing these data to improve our comprehension of the biological effects of high Linear Energy Transfer (LET) radiation^6^. Prior research has conducted exhaustive examinations of these data, employing statistical methods to establish the relationship between neutron exposures and various causes of death. The recent literature has examined the inverse dose-rate effect in carcinogenesis^3^, bystander effects in radon risks^7^, and dose rate effects in heavy-ion carcinogenesis^8^. There are also studies on radiation response age effects^9^, radiation effects on breast cancer risk^10,11^, and the temporal variation of excess mortality rates from solid tumors in irradiated mice.

Most of these analyses used traditional statistical models which operate under stringent assumptions, which may limit the insights they can provide^4,8^. The complexity of radiation effects and interactions between variables generate a need for flexible, robust modeling approaches. To address this need, our study, utilizing data from the Janus archive^6^ on B6CF1 mice exposed to fission neutrons, employs state of the art machine learning, namely the Random Survival Forests (RSF) model^12^, combined with Shapley Additive Explanations (SHAP) values^13^ for interpreting the model. The RSF model, which does not rely on the same strict assumptions as traditional techniques like the Cox regression, provided a more robust analysis of the dose–response relationship for overall survival in relation to fractionation, duration of each fraction, mouse sex, and age at the start of irradiation^12,13^. The RSF model's inherent flexibility, coupled with the interpretability provided by SHAP values, was especially valuable given the potential nonlinear relationships and interactions between various features, which might not be fully captured by traditional models. The combination of techniques that is relatively new to the radiation biology field allows us to perform more nuanced analyses, unraveling complex, nonlinear relationships between variables and generating insights that were previously unattainable^12,13^.

Our study aims to deepen the understanding of the biological effects of high LET radiation. Our analysis approach based on state of the art machine learning techniques aligns with our goal to enhance the body of literature on radiation biology, particularly in high-stakes contexts like space exploration. We believe our findings will not only expand our scientific understanding but also contribute to developing more effective radiation protection strategies^12^.

## Methods

### Data collection

To understand the effects of fission neutrons on mortality, we amassed a comprehensive dataset of B6CF1 mice from the Janus archive^6^, which offers extensive repositories of radiobiological research, providing a rich, reliable source of data. The Janus archive, administered by the U.S. Department of Energy's Argonne National Laboratory, is a leading source of long-term animal studies examining the effects of radiation, including fission neutrons. Importantly, the collected data is at the individual mouse level (instead of averages over experimental groups), which increases the statistical power of potential analyses. Also importantly, the selected mice were allowed to live out their complete life span (instead of planned sacrifice at a specific age), providing more complete information on age-dependent survival curve shapes after various radiation exposures. For our research, we meticulously selected data sets from these archives that contained survival times of individual mice exposed to fission neutrons. We focused on studies that provided data on variables relevant to our study objectives, including the dose and duration of exposure, the sex of the animals, and their age at the start of irradiation. Studies or individual records with incomplete or ambiguous data, or that employed irradiation methods other than fission neutrons, were excluded from our selection to maintain consistency and reliability.

### Mouse selection criteria

In selecting the dataset for our analysis, stringent criteria were applied to ensure the consistency and relevance of the included mice. We only included mice with a species equal to *Mus musculus*, B6CF1strain. Further, only mice with “Autopsied” as their Autopsy Type and a Cause of Death listed as either “Died” or “Sacrifice, moribund” were included, ensuring that the deaths were not due to scheduled sacrifice, transfer to another experiment, cannibalism, or unknown causes. The Radiation type was also controlled, with only “C”, Control, or “N”, neutron radiation types included, thus excluding animals exposed only to gamma radiation. Only neutron doses (without the gamma ray component) were used for the analysis, which should not matter for the conclusions since the gamma ray contribution is a relatively constant fraction of total dose. Moreover, for any entry where the dose was recorded as 0, we automatically set the number of fractions and duration of fraction to 0 as well. This rigorous filtering process allowed us to focus on a consistent and homogenous dataset of around 40,000 mice, thereby enhancing the reliability of our subsequent analyses.

### Data preprocessing

Following the data collection, an extensive preprocessing stage was undertaken to clean and transform the data for our analysis. Several new variables were derived from the existing data, including the number of fractions and the duration of each fraction, which provided additional granularity for our models. All analyses were conducted using R (version 4.3.0) and Python (version 3.10.4, Jupyter Notebooks interface) programming languages. To accommodate our machine learning approaches, we also converted categorical variables into a suitable numeric form. Specifically, the sex of the mice was transformed into a binary numeric variable, with males assigned a value of 1 and females assigned a value of 0.

### Analytical approach

The primary analytical strategies initially considered for this study were Cox Proportional Hazards regression (Cox regression) and Random Survival Forests (RSF). The Cox regression model, implemented using the 'survival' package in R, is a popular method for modeling survival times. This model is semi-parametric, using a parametric form for the covariates and making no assumptions about the baseline hazard function. The key assumption of this model is the proportional hazards assumption, which states that the effects of predictor variables are multiplicatively related to the hazard and are constant over time.

Despite its widespread use, Cox regression may not perform optimally when the proportional hazards assumption is violated or in the presence of complex, non-linear relationships. In our initial analysis, statistical testing and visualization of residuals indicated that the primary assumptions of the Cox regression model were not adequately met, even if the model included several interactions among predictors, signaling the need for an alternative model. As a result, we transitioned to RSF as the preferred analysis method, implemented in Python.

RSF, a machine learning approach, is an extension of the Random Forests (RF) algorithm^12^ for right-censored survival data. The RF algorithm is an extension of the “bagging” (bootstrap aggregation) method as it utilizes both bagging and “feature randomness” to create an uncorrelated forest of decision trees. Bagging is an ensemble method where multiple models are trained on bootstrapped samples of the training data and their predictions are aggregated to yield a more accurate estimate. This approach is commonly used to reduce variance within a noisy data set. Feature randomness refers to the random selection of a subset of features at each node in the tree. This improves variance by reducing correlation between trees. The implementation of RSF follows the same general principles as RF: survival trees are grown using bootstrapped data, random feature selection is used when splitting tree nodes, trees are generally grown deeply, and the survival forest ensemble is calculated by averaging terminal node statistics. The main difference between RSF and RF lies in the splitting rule used to pick the predictor cut-points at the tree branches. In RSF, the splitting rule must specifically account for censoring in right-censored survival data.

The primary advantage of RSF over Cox regression is its non-parametric nature and ability to model complex interactions and non-linear relationships without relying on specific assumptions about the form of these relationships. Both Cox regression and RSF models were used here to analyze the dose–response for overall survival and to discern how this response was modulated by factors such as fractionation, duration of each fraction, sex of the mouse, and age at the onset of irradiation.

### Sensitivity analysis by cross validation

Both Cox regression and RSF models were fitted to the entire dataset. To validate the robustness and generalizability of the models, a tenfold cross-validation was performed. It is a resampling procedure used to evaluate machine learning models on a limited data sample. The procedure has a single parameter called k that refers to the number of groups that a given data sample is to be randomly split into. When k = 10, the data sample is split into 10 groups, and the procedure is called tenfold cross-validation. In tenfold cross-validation, the model is trained on 9 of the 10 groups and tested on the remaining group. This process is repeated 10 times, with each group serving as the test set once. The results of the 10 tests are then averaged to produce an overall performance estimate. This process aimed to test the models' sensitivity to random fluctuations in the data and their capacity to extend beyond the training dataset.

### Interpretation of the RSF model

The interpretation of the RSF model was accomplished using Shapley Additive Explanations (SHAP) values^13^. SHAP values are a powerful tool for interpreting machine learning models. Rooted in cooperative game theory, they offer a unified measure of feature importance. By attributing the change in expected model prediction to each feature in the presence of all possible combinations of features, SHAP values allow for a fair allocation of contributions from each feature. Importantly, this includes considering interaction effects among features. Thus, SHAP values not only provide insights into the relative importance of variables but also illuminate the nature of their effects on the model's predictions. They strike a balance between maintaining the accuracy of complex models while providing the interpretability often associated with simpler models. The SHAP value formula is summarized below. *F* represents the number of features (predictors) in the model, *S* represents a subset of these features, *v* is the function that generates the value of the model’s prediction based on the features, *i* is the index of the feature of interest, and SHAP_i_ is the SHAP value of feature *i*:

$${SHAP}_{i}=\frac{1}{\left|F\right|}\sum_{S\subseteq F-i}[{(\begin{array}{c}\left|F\right|-1\\ \left|S\right|\end{array})}^{-1}(v\left({S}_{i}\right)-v(S))]$$

The terms in this equation have the following interpretations. $1/|F|$ is a scaling factor. $S\subseteq F-i$ indicates that the feature of interest (*i*) is excluded from the set for the current calculation. $${(\begin{array}{c}\left|F\right|-1\\ \left|S\right|\end{array})}^{-1}$$ represents the number of groups of size |*S*| formed from |*F*|-1 features. $v\left({S}_{i}\right)-v(S)$ is the marginal value of adding feature *i* to the set.

By employing SHAP values, we were able to delve into an in-depth understanding of how each variable influences the survival rates of mice exposed to fission neutrons. This nuanced approach to model interpretation thus afforded a comprehensive understanding of our data and the relationships within. SHAP values were calculated on the testing portion of the data set: 20% of the data, selected randomly.

### Interpreting SHAP values for RSF risk scores

In the specific context of the RSF model implemented in Python, the SHAP values represent risk scores, which correspond to the expected number of events for one particular terminal node of each tree in the forest. By attributing these risk scores to each feature, we can gain insights into how each feature influences the outcome of interest—in this case, the survival rates of mice exposed to fission neutrons. While the absolute units of the SHAP values in this case do not directly correspond to survival times, they allow us to rank features by importance and to discern how each feature contributes, positively or negatively, to the risk of event occurrence. To improve interpretability, we normalized the SHAP values as follows, where *SHAP* is the SHAP value of a particular feature, *B* is the baseline SHAP value across all features (in a sense, an intercept), and *SHAP*_*n*_ is the normalized value:

$$SHA{P}_{n}=\frac{SHAP+B}{B}-1$$

This approach has the advantage of removing the arbitrary risk score units and converting the SHAP values to a relative scale, which can be interpreted as a relative effect on mortality risk. Therefore, *SHAP*_*n*_ for a given feature such as dose provides a convenient metric for quantifying and visualizing the effect of that feature on mortality predicted by the RSF model.

For improved visualization, for each feature the median *SHAP*_*n*_ value corresponding to the feature value of 0 was subtracted, so that the *SHAP*_*n*_ responses start at the origin. Second order polynomial quantile regression fits to the median of the *SHAP*_*n*_ values were fitted for each feature to visualize simplified quantitative trends for how each feature contributed to mortality predictions.

### Focused analysis on low-dose exposure

In order to examine in more detail the effects of neutrons at low doses (< 20 cGy), which are relevant for space travel and many occupational and accidental exposure scenarios, we isolated the subset of our data which fell within this dose range and then generated SHAP value graphs specifically for this segment of the data. This allowed us to more precisely examine the impact of dose, dose fractionation, and fraction duration on these low-dose exposures.

SHAP value plots were produced for each of these three variables (dose, dose fractionation, and duration), offering visual interpretations of the influence these factors exert on the model’s output within the low-dose exposure scenario. This additional layer of analysis provided enhanced depth and specificity to our findings, with implications for risk assessment and management strategies in low-dose radiation environments.

## Results

Our dataset, curated from the Janus archive, contained survival times of a total of 21,308 individual mice exposed to fission neutrons. The dataset consisted of variables including the sex of the mice, age at the start of irradiation (ranging from 93 to 519 days), age at death (ranging from 113 to 1517 days), neutron dose (ranging from 0 to 323.79 cGy), number of fractions (ranging from 0 to 180), and duration of each fraction (ranging from 0 to 480 min). The summary statistics, presented in Table 1 and in Supplementary Table 1, offer an overview of the distribution across these key variables and form the basis of our subsequent analysis.

**Table 1:** Summary of each variable in the analyzed data set.

	Age.at.Death	Sex	Experiment	Treatment.Age	Dose	Number.of.Fractions	Duration.of.Fraction
Count	21,308.000000	21,308.000000	21,308.000000	21,308.000000	21,308.000000	21,308.000000	21,308.000000
Mean	895.373005	0.484560	6.598555	128.375399	56.177335	17.844425	27.041017
Std	214.676305	0.499773	4.118282	69.358903	79.745498	26.655991	49.513703
Min	113.000000	0.000000	2.000000	93.000000	0.000000	0.000000	0.000000
25%	758.000000	0.000000	3.000000	108.000000	0.000000	0.000000	0.000000
50%	907.000000	0.000000	7.000000	113.000000	18.840000	1.000000	20.000000
75%	1045.000000	1.000000	9.000000	116.000000	75.360000	24.000000	20.000000
Max	1517.000000	1.000000	13.000000	519.000000	323.790000	180.000000	480.000000

The variable meanings are: Age.at.Death = age in days when the mouse died; Sex = 0 for females and 1 for males; Experiment = integer indicator for Janus study arm (not used in the analysis); Treatment.Age = mouse age in days at the start of irradiation; Dose = neutron dose in cGy; Number.of.Fractions = fractionation of the dose; Duration.of.Fraction = duration of each fraction in minutes. The summary metrics are: count = total number of values for each variable; mean = average; std = standard deviation; min = minimum; 25% = 25th percentile; 50% = 50th percentile; 75% = 75th percentile; max = maximum.

**Figure 1:**
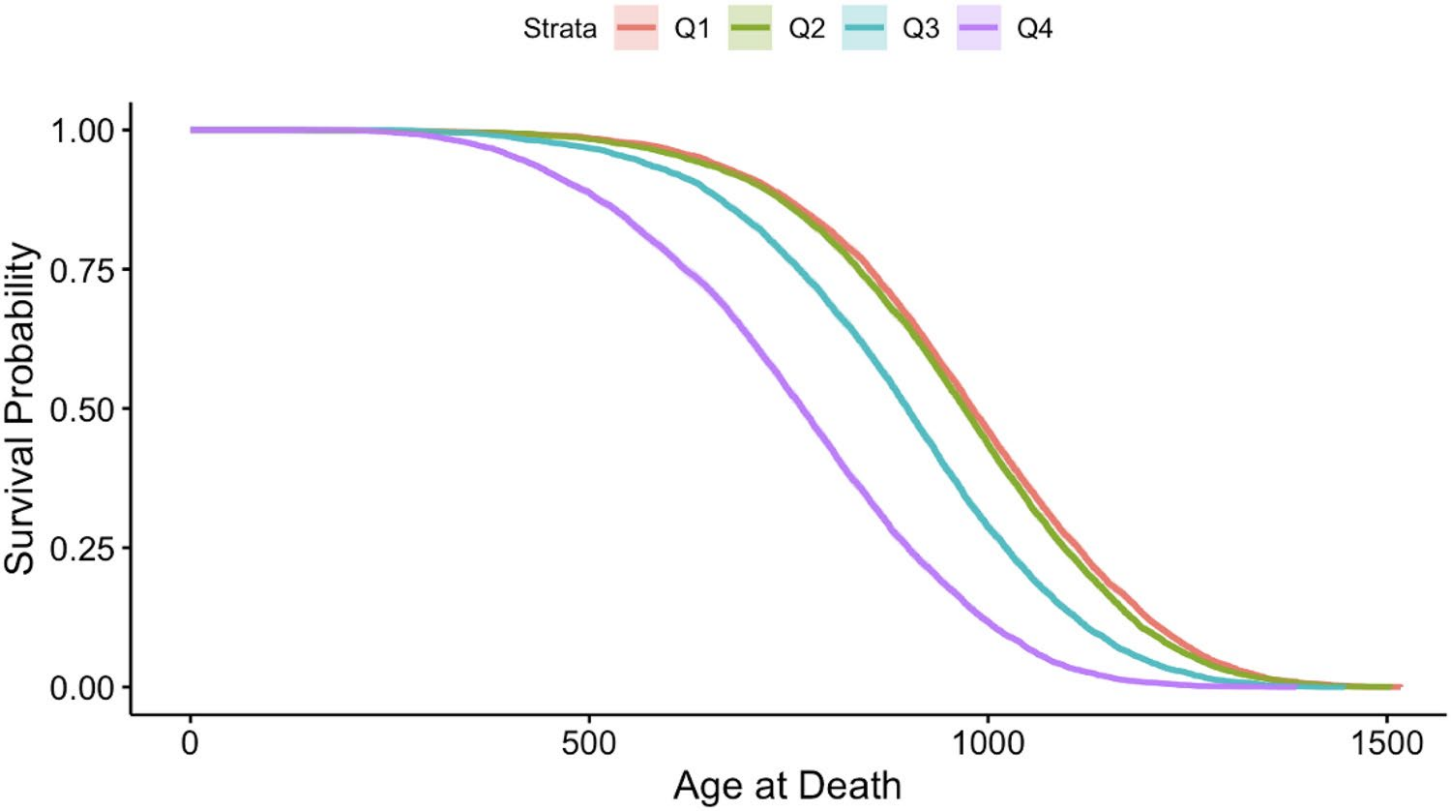
Kaplan–Meier survival curves for each dose quartile (quartiles represented by labels Q1-Q4). Q1 = 0.00, Q2 = 18.84, Q3 = 75.36, Q4 = 323.79 cGy. Age at death is in days.

**Figure 2:**
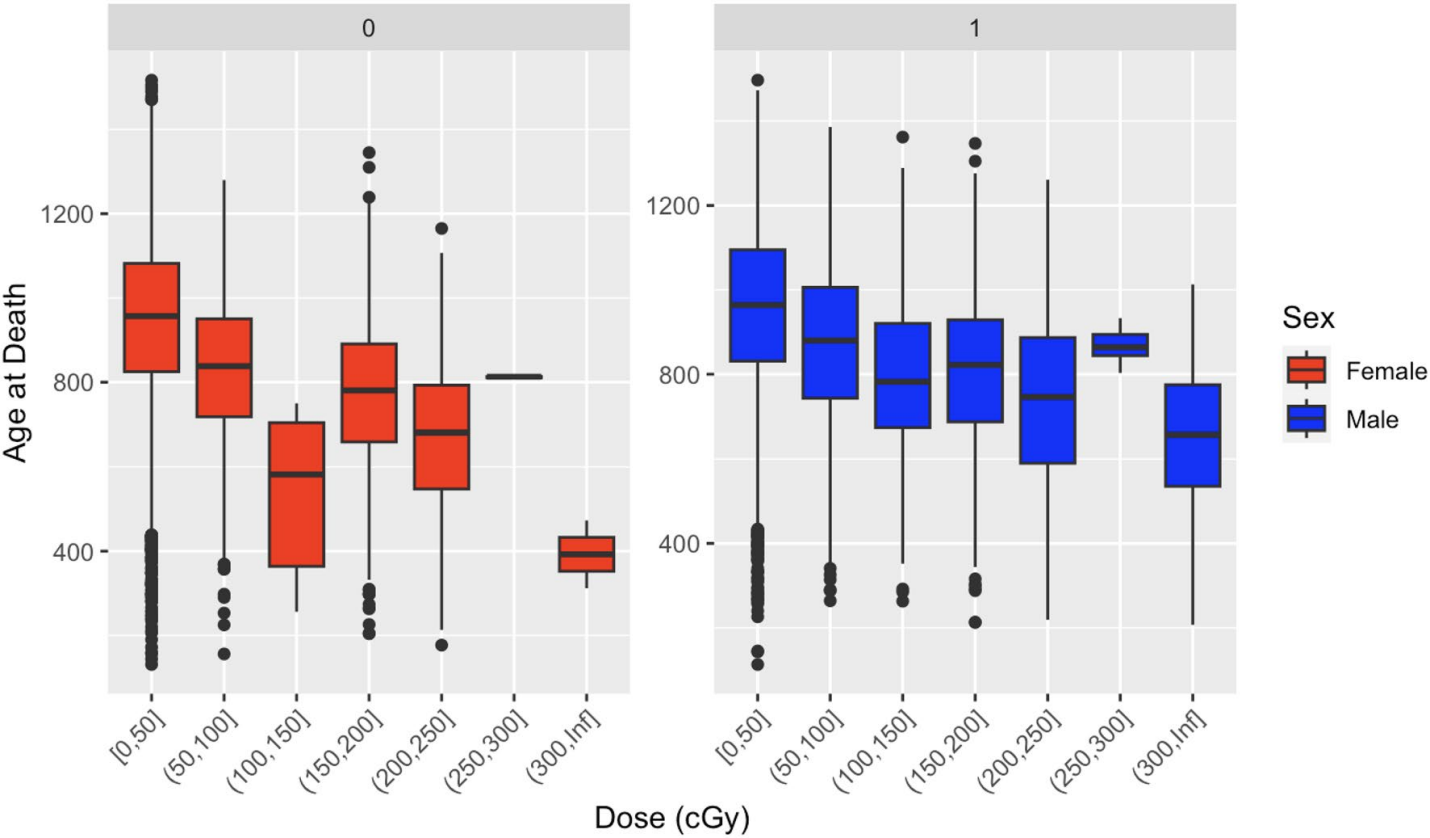
Boxplot of Age at Death by Dose and Sex. Female is on the left (red), and male is on the right (blue). The central line within each box represents the median value. The box boundaries denote the interquartile range (IQR), illustrating the middle 50% of the data. Whiskers extend from the box to the minimum and maximum values within 1.5*IQR. Individual points beyond the whiskers represent outliers.

To visualize the dose-dependent effect of neutron exposure on mouse survival, we plotted the Kaplan–Meier survival curves for each dose quartile (Fig. 1). These curves clearly show that survival time was reduced by increasing neutron dose, and that the shapes of the survival curves also changed as function of dose. Boxplots showing how the distribution of mouse ages at death changed as function of increasing neutron dose are shown in Fig. 2. They also show a decreasing tend with increasing dose, except for the highest dose bin where the exposures were highly fractionated (Supplementary Table 1).

We initiated our analysis by performing Cox regression, since it is often the default method for survival analysis due to its simplicity and interpretability. The Cox model was a type of survival analysis model, providing us with an estimated hazard ratio for each predictor variable. The hazard ratio can be thought of as a risk multiplier. For instance, if the hazard ratio for a particular variable, say dose, is 1.5, this implies that for every unit increase in dose, the hazard (or risk) of the event (in our case, death) increases by 50%. Importantly, the Cox model assumes that these hazard ratios are constant over time, which is a key consideration when interpreting our results.

We initially fitted a simple Cox model variant with only main effects for the variables of interest: Dose, Number.of.Fractions, Duration.of.Fraction, Sex and Treatment.Age. However, this model failed the cox.zph test for the proportional hazards assumption (Supplementary Fig. 1A). We then fitted a more complex Cox model version with interaction terms, but the proportional hazards assumption was still significantly violated for every variable, except for Number.of.Fractions (Supplementary Fig. 1B). Plots of the Martingale residuals for the Cox model without interaction terms (defined as the difference between the observed number of events and the expected number of events under the fitted model for each individual), shown in Supplementary Figs. 2–5, suggest that the residuals tend to be asymmetrically negative. This finding implies that the fitted survival curves produced by the Cox model, while starting and ending at the same points as the observed data, were consistently slightly above the observed survival curve values. This demonstrated that the Cox model systematically overestimated the survival times across different levels of the covariates, hinting at a lack of good fit.

Scaled Schoenfeld residuals for different variables in the Cox model without interaction terms are plotted as function of time in Supplementary Figs. 6–10. These plots show how the estimated regression coefficients change over time. Flat patterns with no time dependence suggest that the proportional hazards assumption holds, whereas deviations indicate assumption violations, as observed here (*e.g.* in Supplementary Fig. 8).

The concordance (c-index), which quantifies the agreement between predicted and observed event times, was used to assess model performance quantitatively. The c-index represents the global assessment of the model discrimination power: this is the model’s ability to correctly provide a reliable ranking of the survival times based on the individual risk scores. It is defined as the ratio of correctly ordered (concordant) pairs to comparable pairs of observed and model-predicted survival times.

The c-index value obtained from the Cox regression without interaction terms is 0.599, displaying an improvement from random guessing, but not a very dramatic one. The performance was further assessed during tenfold cross-validation (Supplementary Fig. 11). The range of c-index for the training data was from 0.583 to 0.599, with an average value of approximately 0.589. The range of c-index for the test data was from 0.550 to 0.637, with an average value of approximately 0.588. These statistics reflect consistent model performance across portions of the data set.

These observations of limitations of the Cox model on the data set analyzed here, such as violation of the proportional hazards assumption and asymmetric residuals, underscored the necessity of adopting a more flexible modeling approach to better represent the intricate relationships within our data. A good candidate for this role is the Random Survival Forest (RSF) algorithm, which applies the random forest concept developed by the famous statistician Leo Breiman^12^ to survival data. It works by building multiple survival trees on various sub-samples of the data set and using averaging to improve predictive accuracy and control over-fitting. Each tree is grown by taking into account censoring when choosing the best split at each node. The final prediction for a new observation is obtained by averaging the predictions from all the trees in the forest. RSF is useful here because it can capture complex relationships between the predictors and survival without requiring strict assumptions or shapes for these dependences.

The RSF model was implemented in Python 3 with hyperparameter values of n_estimators = 25, min_samples_leaf = 22, and min_samples_split = 2 tuned by a grid search procedure. n_estimators refers to the number of trees in the forest, min_samples_split specifies the minimum number of samples required to split an internal node, and min_samples_leaf specifies the minimum number of samples required to be at a leaf node. This model achieved c-index values of 0.6550 and 0.6484 on the training (70% of the data) and testing (30% of the data) portions of the data, respectively. Furthermore, a tenfold cross-validation approach revealed very similar c-index values with an average of 0.6551 on the training folds and 0.6499 on the validation folds, exhibiting the model's robustness and minimal overfitting with a mean difference between the testing and training c-indexes of only − 0.0052.

**Figure 3:**
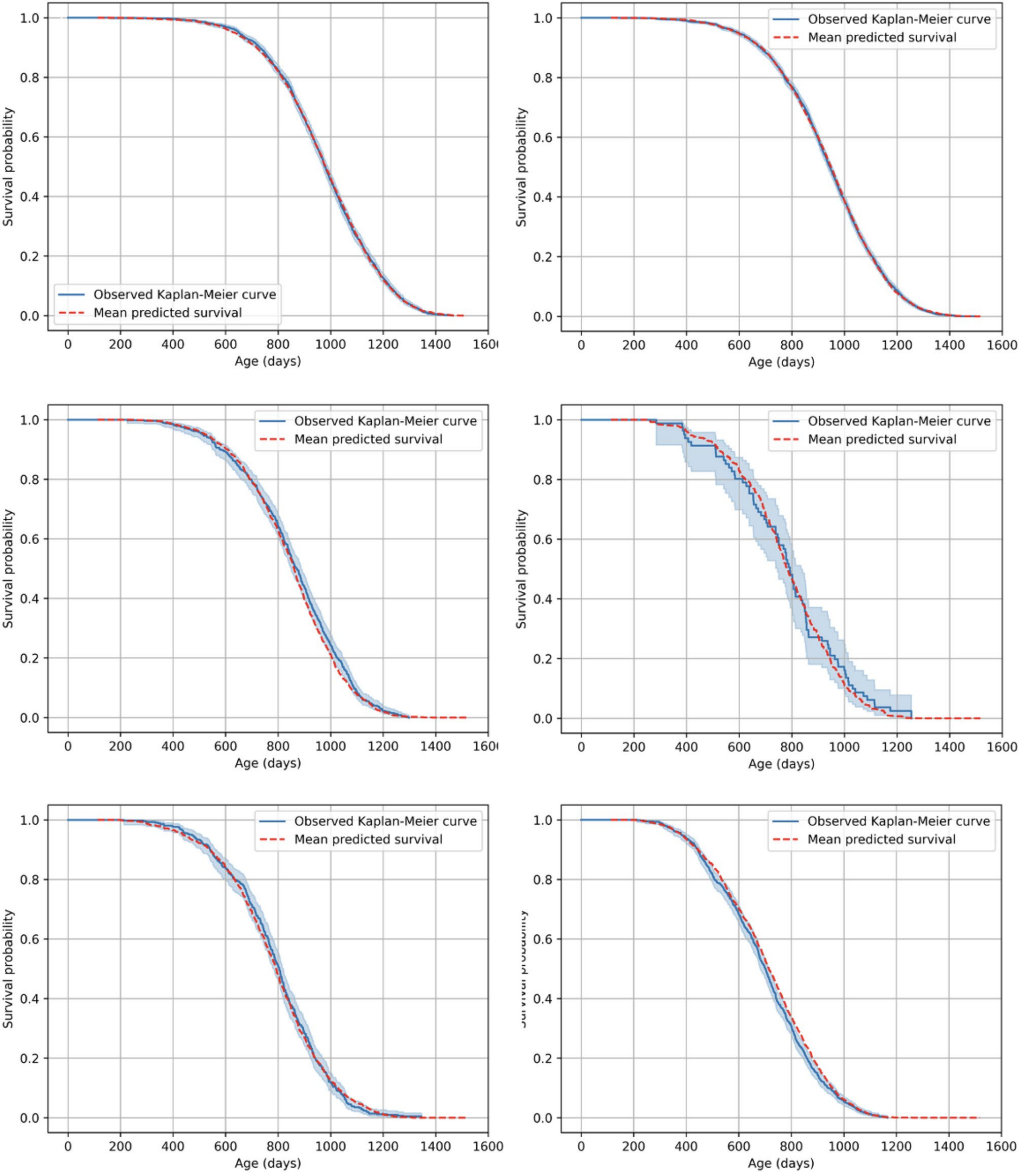
Survival Plots for the Random Survival Forest Model (red curves) versus observed Kaplan Meier curves (blue curves). Plots numbered 1–6 represent subsets of the testing data by dose (in units of cGy): Plot 1 = dose = 0; 2 = dose > 0 and ≤ 50 cGy; 3 = dose > 50 and ≤ 100 cGy; 4 = dose > 100 and ≤ 150 cGy; 5 = dose > 150 and ≤ 200 cGy; 6 = dose > 200 cGy. The blue shaded region represents the 95% confidence interval band for observed survival.

These c-index values attained by RSF were somewhat higher than those for the Cox model mentioned above. RSF provided visually good fits to the testing data, which were shown in Fig. 3. Mean RSF-predicted survival curves for groups of mice exposed to different neutron doses were very close to the corresponding observed Kaplan–Meier survival curves for these groups, at both low and high doses (Fig. 3). This close correspondence suggests that RSF was able to capture the main dose response trends in the data, although variations in survival for individual mice in each group were considerable and likely explain why the c-index did not approach unity.

The tradeoff for increased flexibility and fewer assumptions in the RSF model, relative to the simple Cox model, involves somewhat less straightforward interpretability. RSF does not generate a fitted coefficient for each predictor variable, but there are state of the art tools to interpret its behaviors and explain how the predictors influence the predicted outcome. RSF interpretation is aided by the use of SHAP (SHapley Additive exPlanations) values. These values measure the contribution of each feature (predictor variable) to the predicted outcome for each individual in our testing dataset, accounting for all possible orders in which the features can contribute to the model.

**Figure 4:**
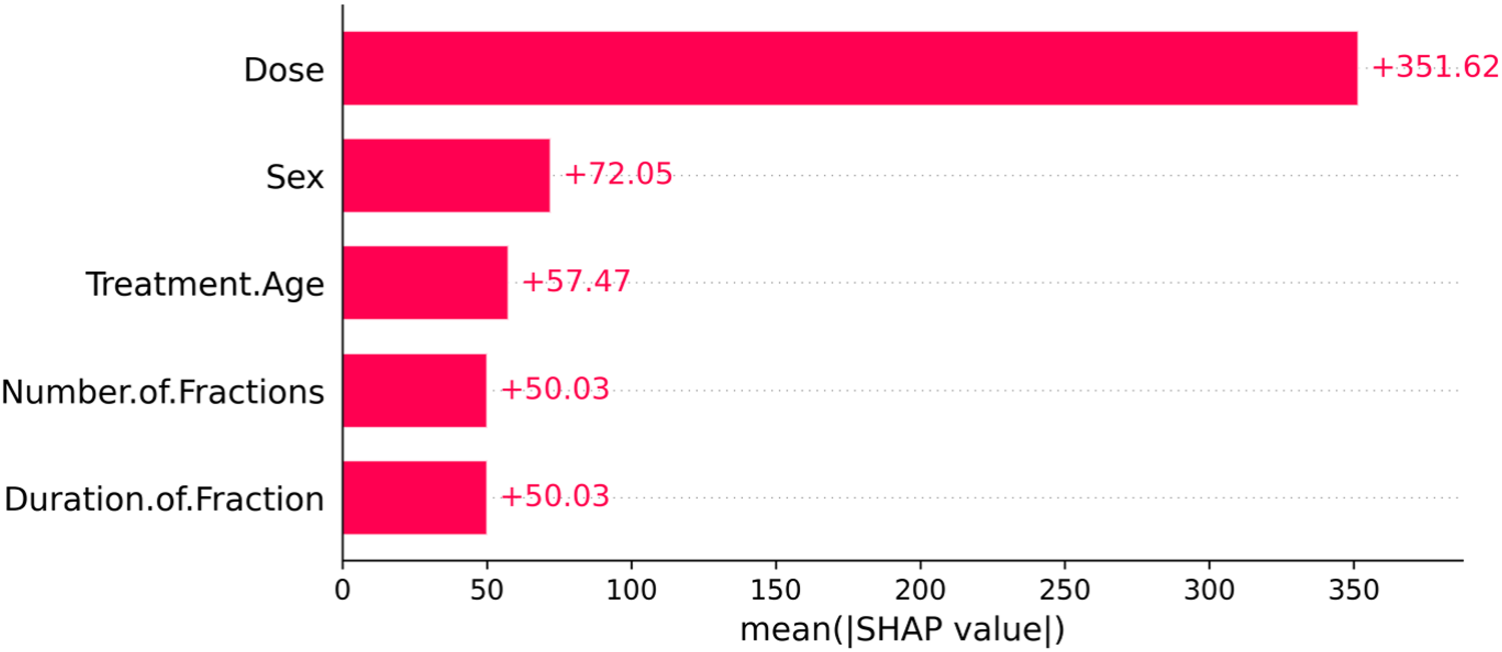
Mean absolute value SHAP values for each feature in the RSF model. In this and the following figures, SHAP value units are proportional to death risk scores.

Figure 4 shows mean absolute values of the SHAP values for each feature, and suggested that the most important features overall were neutron dose and sex. Treatment age (age at the start of irradiation), number and duration of fractions had somewhat smaller effects on model predictions (Fig. 4). These findings are generally in line with expectations.

**Figure 5:**
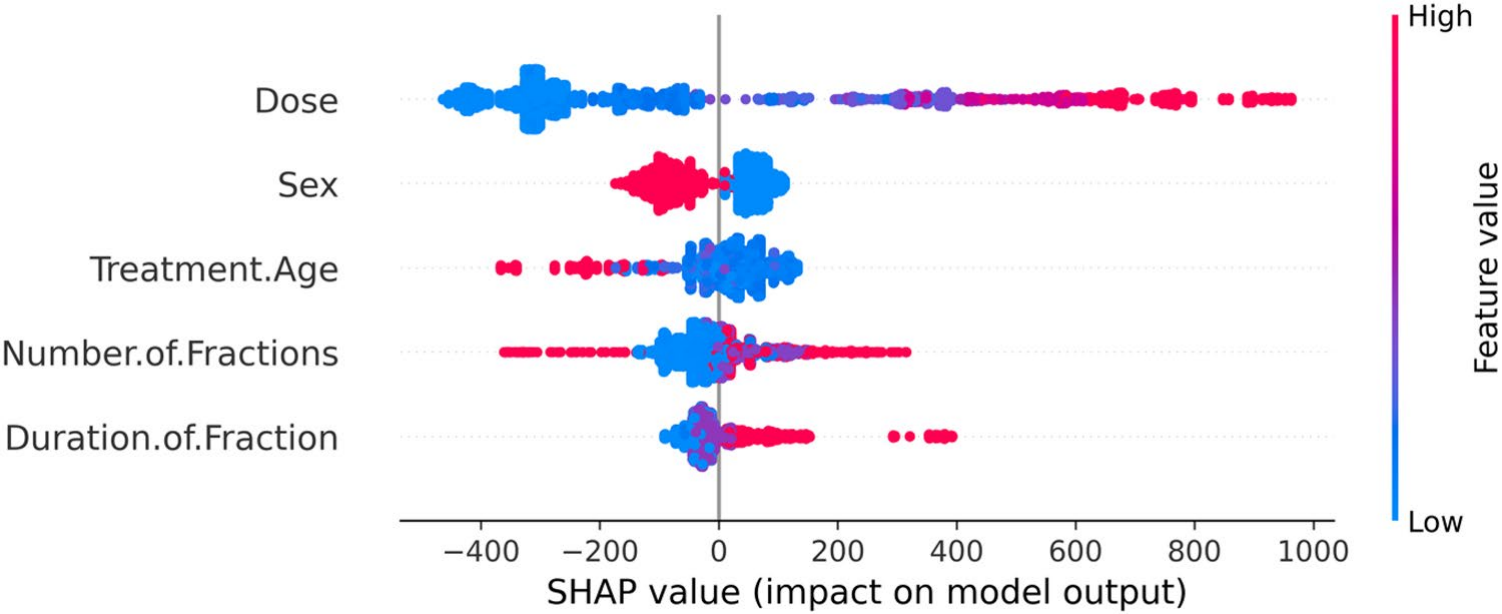
Summary of individual SHAP values. The color of the points indicates the value of the feature for that observation, with warm colors typically representing higher values and cool colors representing lower values. The SHAP value scale indicates the effect on model predictions: a positive SHAP value implies an increased risk of death, while a negative value suggests a reduced risk.

To examine not only the global average SHAP values for each feature, but to focus on SHAP value behavior at the individual mouse level, we generated detailed SHAP plots discussed below. A summary of individual SHAP values is shown in Fig. 5. The color of the points indicates the value of the feature for that observation, with warm colors typically representing higher values and cool colors representing lower values. The SHAP value scale indicates the effect on model predictions: a positive SHAP value implies an increased risk of death, while a negative value suggests a reduced risk. For instance, the SHAP plot for the variable Dose showed that higher doses (represented by warmer colored points) were generally found towards the right side of the plot, and this suggests that higher radiation doses increase the risk of death. Similarly, for the number of fractions variable, larger fraction numbers (warmer colors) were associated with lower SHAP values (towards the left of the plot), suggesting that fractionation decreases the risk. Such interpretations provide valuable insights into the influences of various radiation-related factors on survival.

**Figure 6:**
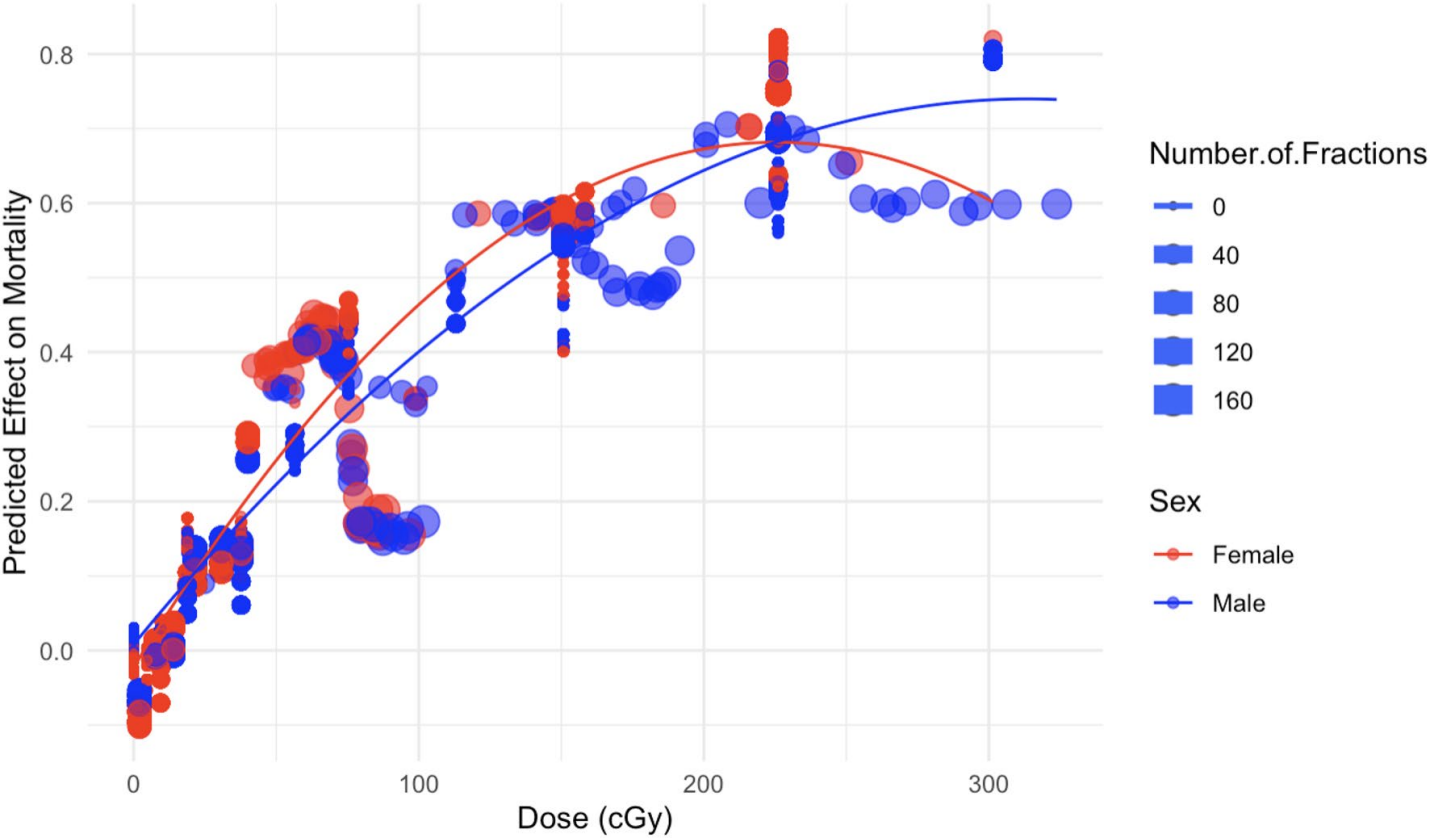
The effect of dose on predicted mouse mortality. In this and the following figures, circles indicate normalized SHAP values for individual mice, and curves represent second order (linear quadratic) polynomial quantile regression fits to the median (50th percentile). Here larger circles represent fractionated exposures, and smaller circles represent single-fraction exposures.

**Figure 7:**
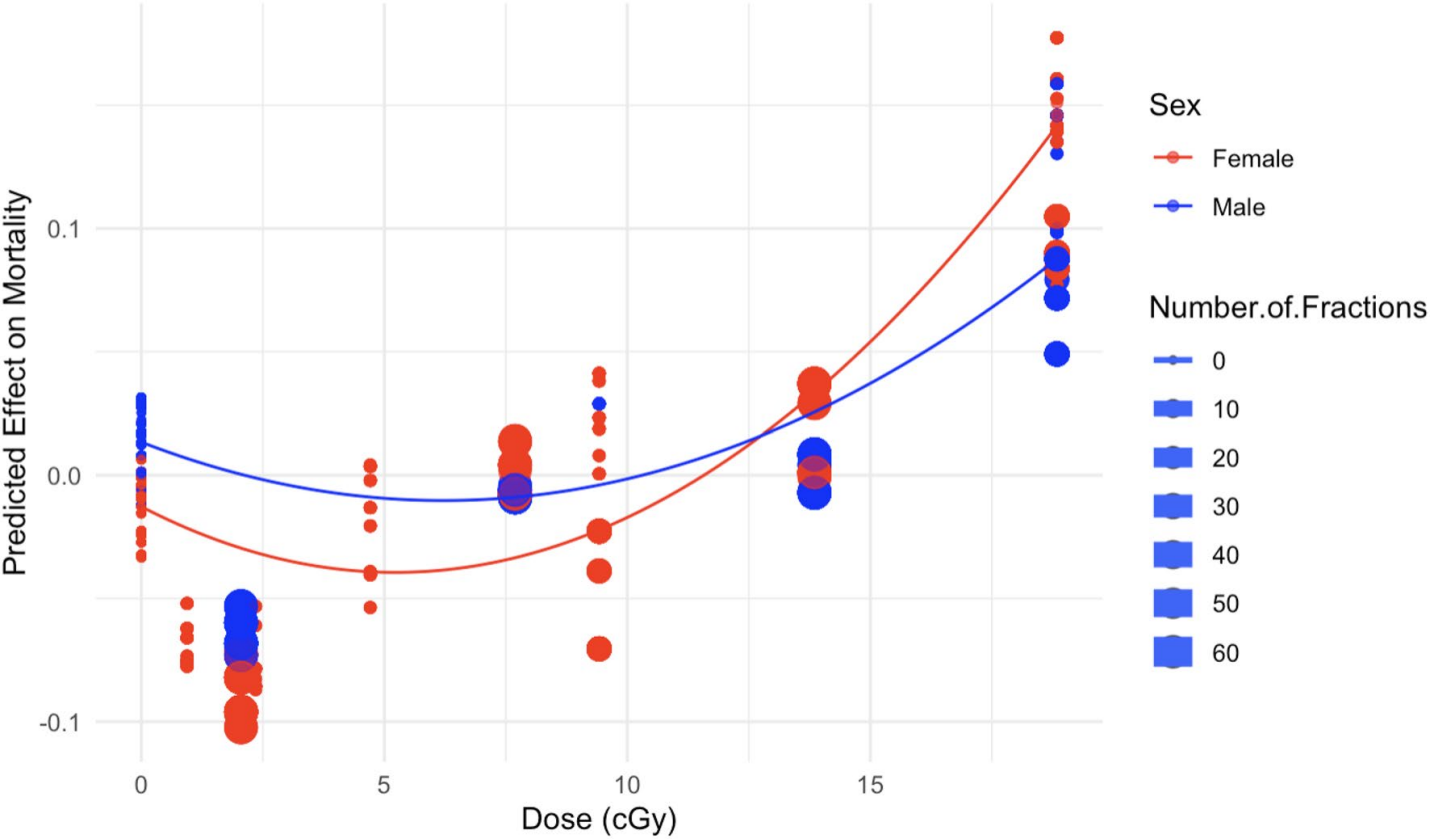
The effect of dose on predicted mouse mortality, but for the subset of data with dose ≤ 20 cGy.

**Figure 8:**
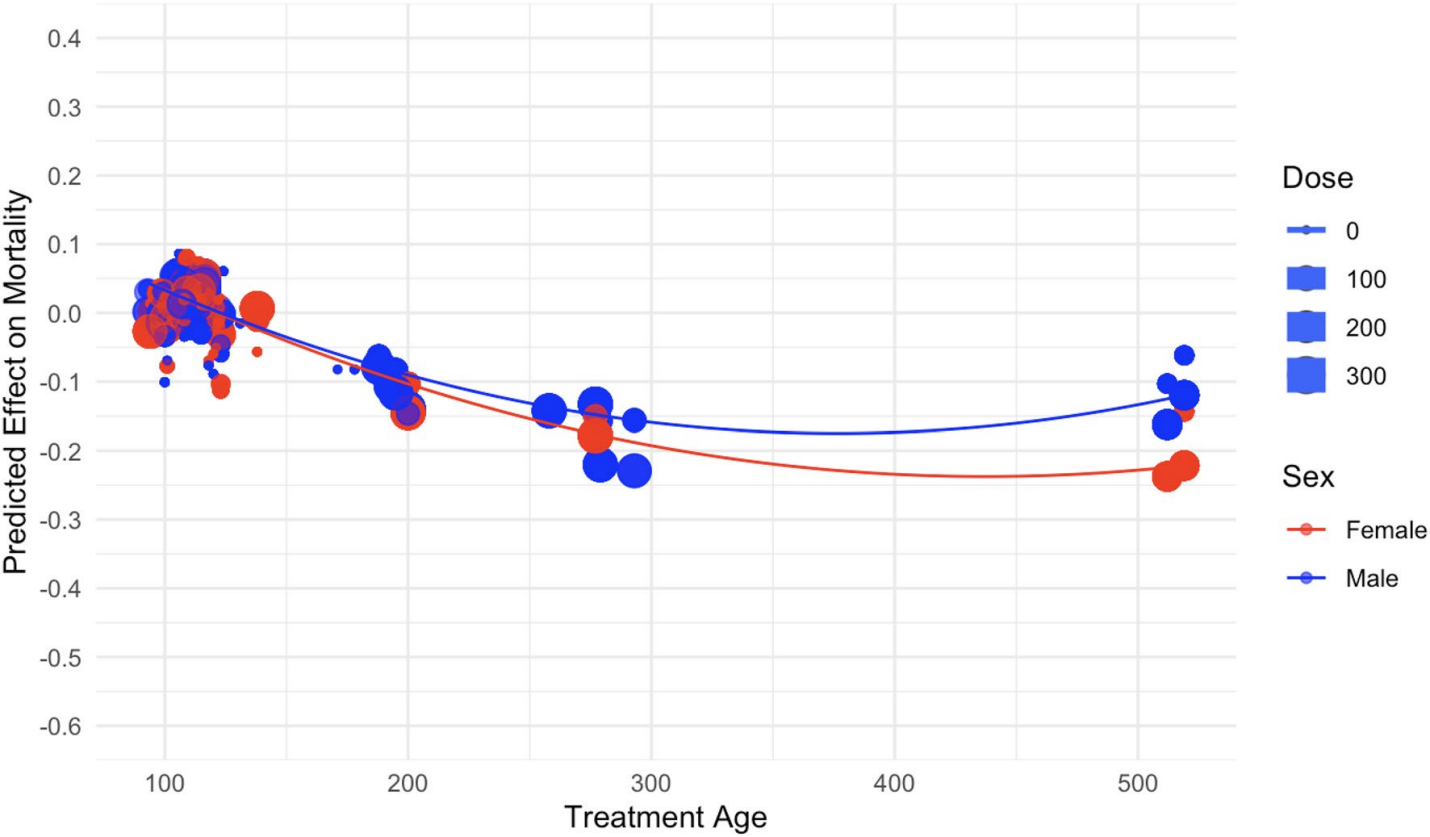
The effect of treatment age (days) on predicted mouse mortality.

**Figure 9:**
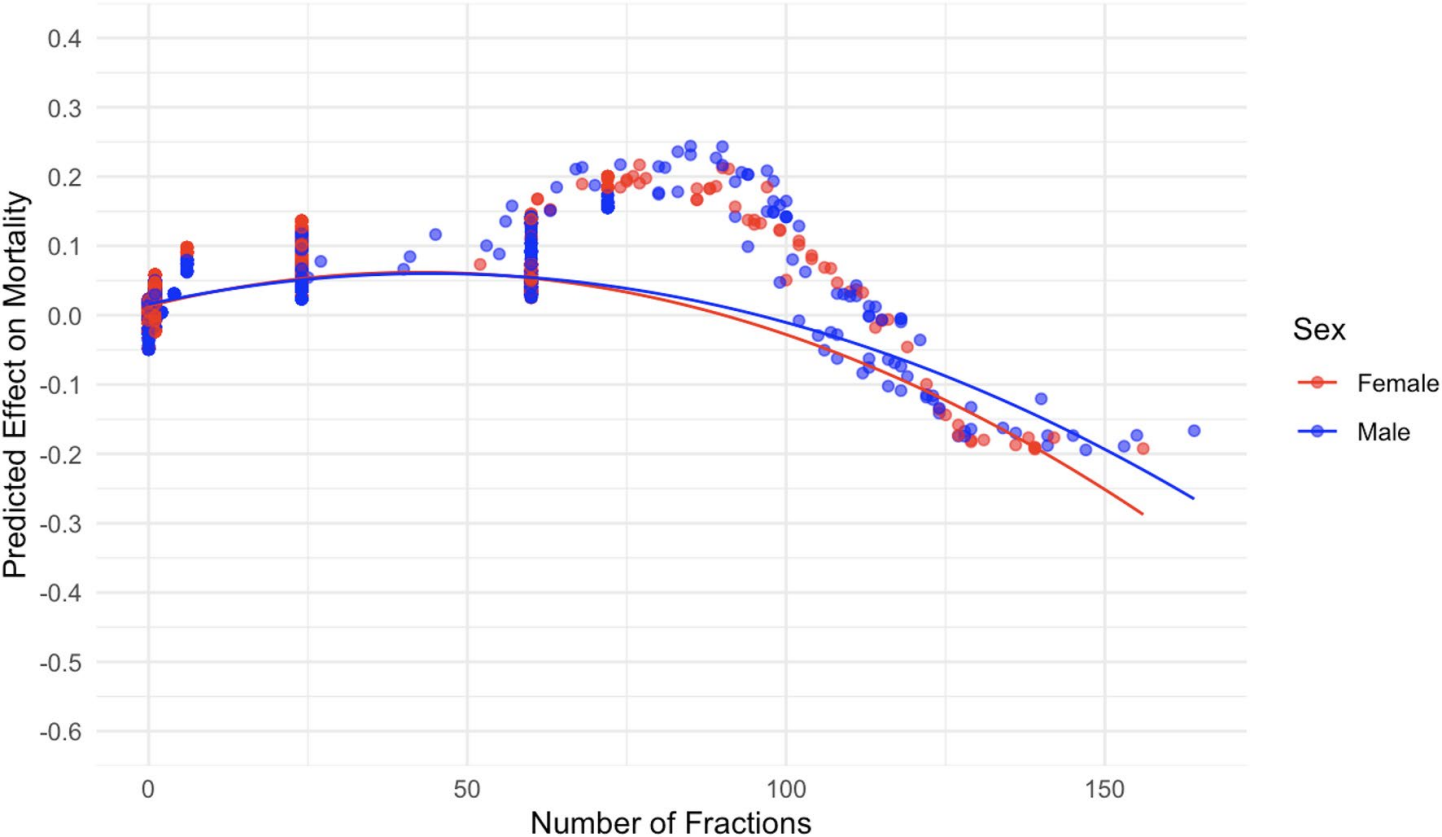
The effect of number of fractions on predicted mouse mortality.

**Figure 10:**
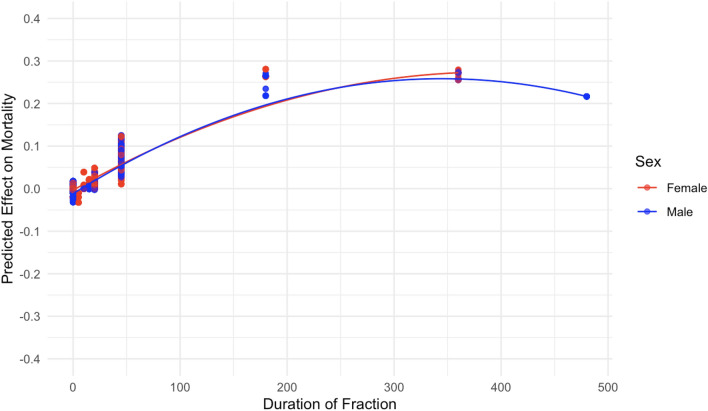
The effect of duration of fraction (minutes) on predicted mouse mortality.

**Figure 11:**
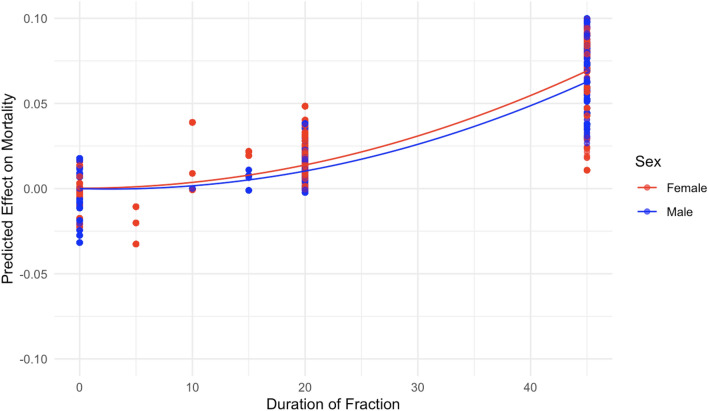
The effect of duration of fraction (minutes) on predicted mouse mortality, but for the subset of data < 50 min/fraction.

More detailed normalized SHAP plots for individual variables, which provide a convenient metric for quantifying and visualizing the effect of each selected variable on mortality predicted by the RSF model, are shown in Figs. 6, 7, 8, 9, 10 and 11. The relationship between neutron dose and its normalized SHAP values (Figs. 6 and 7) is of particular interest here because this represents a dose response. This relationship is displayed for both male and female mice over all of the dose range (Fig. 6), and also zoomed in at low doses < 20 cGy (Fig. 7). In these SHAP plots, each point on the plot represents a single observation (mouse). The x-axis shows the feature value—dose in this case. The y-axis shows the corresponding normalized SHAP value.

Considering the degree of scatter between normalized SHAP values for individual mice, the shape of the dose response has potential upward curvature at low doses < 20 cGy (Fig. 7), the becomes close to linear at medium doses of about 20–150 cGy, with some downward curvature or saturation at higher doses (Fig. 6). Parameters for the best-fit polynomial curves are shown in Table 2. The differences in dose responses between different dose fractionations and sexes are not dramatic (Figs. 6 and 7), as also indicated by the relatively small mean absolute SHAP values for these variables, compared with dose (Fig. 4).

**Table 2:** Polynomial quantile regression fit coefficients for the relationship between each studied variable (over its full range in the data) and its median normalized SHAP values.

Variable <chr>	Dose <dbl>	Treatment_Age <dbl>	Number_of_Fractions <dbl>	Durations_of_Fraction <dbl>
Intercept	− 0.16870	0.00378	− 0.00467	0.00422
Linear	4.21112	− 2.93994	1.51733	3.51428
Quadratic	− 0.41290	1.51662	− 1.84624	− 1.65247
SE_Intercept	0.00068	0.00042	0.00041	0.00023
SE_Linear	0.04547	0.03366	0.03315	0.01857
SE_Quadratic	0.04547	0.03366	0.03315	0.01857

The table outlines the Intercept, Linear, and Quadratic coefficients, representing the baseline SHAP value, primary linear relationship, and any non-linear trends. Standard errors (SE) are provided for each coefficient.

The normalized SHAP values as function of treatment age are shown in Fig. 8. Older age at the start of irradiation generally tended to reduce the risk of death slightly, which is consistent with the literature^9–11^. This pattern is often explained by reduced numbers of actively proliferating stem and progenitor cells with increasing age, and by the reduced time that any of these cells mutated by radiation would have to develop into tumorigenic lineages before the organism reaches old age. This age effect was somewhat modulated by sex, but not dramatically by dose fractionation (Fig. 8).

The relationship between number of fractions and corresponding SHAP values (Fig. 9) suggested a nonlinear relationship that was not well fitted by a second order polynomial. Interestingly, the maximum effect was predicted to occur not for single-fraction exposures, but for regimens with 75–100 fractions, whereas at even higher numbers of fractions the effect declines (Fig. 9). This finding may represent an inverse dose rate/protraction effect, which is known to occur for some densely ionizing radiation exposures and endpoints such as neutron-induced or radon-induced carcinogenesis^2,4,14,15^.

Patterns related to the duration of each fraction are shown in Figs. 10 and 11. Increasing the duration of each fraction tended to increase mortality, which may be consistent with an inverse dose rate effect mentioned earlier. In other words, protraction of the neutron exposure by increasing the number of fractions and/or the duration of each fraction tended to increase (rather than decrease) mouse mortality, compared with single-fraction irradiation.

All possible pairwise Pearson correlations between the variables of interest and/or their SHAP values were investigated in Supplementary Figs. 12–15. Such analyses are informative because they visualize which features and SHAP values are correlated to each other and thus contribute in similar (or opposite) ways to model predictions. This applies in cases where some features are continuous, whereas others are binary or categorical (*e.g.* sex in this case). The results suggest that the magnitudes of pairwise correlations were generally not very strong, <|0.5|, so that the contributions of individual variables were not strongly collinear (Supplementary Fig. 12).

SHAP values tended to be strongly correlated only to their corresponding features (e.g. SHAP for Dose with Dose, SHAP for Sex with Sex), but not with other variables Supplementary Figs. 13–15. However, there were some exceptions. Specifically, as indicated in Supplementary Fig. 14, Norm.SHAP_Number.of.Fractions showed a relatively strong correlation with Dose (correlation coefficient 0.415) compared to its correlation with Number.of.Fractions (0.403). This suggests that the influence of neutron dose on the model's output was more pronounced than the direct relationship between the number of fractions and their corresponding SHAP values. Hence, care should be taken in interpreting SHAP values as uniquely associated with their corresponding variables, as interactions between different features can also influence these importance metrics. Overall, these findings imply that the features were generally not redundant to each other in their contributions to RSF model predictions.

## Discussion

Our analysis of the large data set of neutron-irradiated mice was intended to provide insights into the shape of the dose–response on mortality, and assess the effects of fractionation, protraction, age and sex. Attempts to use Cox regression to model these dependences showed that the proportional hazards and linearity assumptions of this method were not supported by the data. Therefore, the more flexible RSF algorithm, which does not rely on strict assumptions, was implemented. Interpretation of RSF using SHAP values indeed suggested that many of the effects were non-linear. These measures provided clear insights into the relative importance of predictor variables and offered a reliable means of deciphering the impact of different variables on the overall model predictions.

From an inspection of the SHAP graphs for the RSF model, we observe a dose–response relationship that appears slightly curving upwards at low doses < 20 cGy, not too different from linearity at intermediate doses of about 20–150 cGy, with some evidence of saturation or downturn at high doses (Figs. 6 and 7). Summaries of the polynomial fitted curves for these relationships are shown in Table 2. The effects of fractionation/protraction of neutron exposure are consistent with an inverse dose rate effect (Figs. 9, 10 and 11). Older age at the start of irradiation tended to reduce mortality (Fig. 8). Sex also had an influence on model predictions, with lower mortality predicted for males (Figs. 4 and 5). The sex effect was not strongly correlated with the effect of neutron dose (Supplementary Figs. 13–15).

Our findings deliver a nuanced view of survival data for mice exposed to fission neutrons, one that surpasses the level of detail offered by previous analyses. The RSF model's ability to model complex interactions and nonlinear relationships between variables accentuates its potential as a potent tool in future radiobiology research. The results of our study corroborate existing literature on the influences of fractionation and age at treatment onset on survival. More significantly, our findings offer new insights into the effects of mouse sex and duration of each fraction on survival. The complex interplay of these factors shapes a richer understanding of survival mechanisms under neutron radiation, suggesting future lines of investigation. The implications of our research are broad and significant. Our work encourages a pivot towards machine learning methods like the RSF model in radiobiology, given its demonstrated robustness and flexibility. Furthermore, insights gleaned about the influence of fractionation, age, sex, and fraction duration on survival times can inform experimental design and interpretation in space radiation research. In addition, our results could guide personalized treatment planning in human radiation therapy, presenting potential clinical applications.

### Limitations of the RSF model

This study, while advancing our understanding of the effects of neutron radiation using the Random Survival Forests (RSF) model, encounters certain limitations:

1. Model Complexity: The RSF model's complexity can be a double-edged sword. While it allows for capturing non-linear relationships and interactions among variables, this complexity also makes the model less interpretable compared to simpler models like Cox regression. This could pose challenges in explaining model decisions in simpler terms, especially to audiences not familiar with advanced machine learning techniques.
2. Model Validation Challenges: Validating RSF models presents unique challenges, mainly due to their non-parametric nature. The model’s performance, as indicated by the c-index, shows improvement over traditional models, but it does not fully capture the variance in individual survival times. Additionally, RSF models can be prone to overfitting, especially in datasets with numerous features or complex interactions.
3. Generalizability: While RSF offers robust insights for the specific dataset of B6CF1 mice, the generalizability of these findings to different species, radiation types, or exposure scenarios may be limited. Care must be taken when extrapolating these results beyond the studied context.
4. Potential for Future Improvements: Future research could focus on enhancing the interpretability of RSF models and developing more sophisticated validation techniques. This would further strengthen the model's utility in complex survival data analysis and broaden its applicability in radiation biology and other fields.

These limitations notwithstanding, the RSF model represents a significant step forward in analyzing complex survival data and offers a promising avenue for future research in radiation effects and related areas.

## Conclusion

Our study represents a thorough analysis of survival data of large numbers of mice exposed to neutron radiation, uncovering detailed insights that enrich the existing body of knowledge in radiobiology. Moving beyond traditional statistical approaches, our use of innovative analytical methods like RSF and SHAP has enabled us to extract sophisticated understandings of complex radiobiological data. This approach has led to a more nuanced understanding of how radiation affects overall survival, revealing complex, nonlinear relationships between variables, and enhancing the model's robustness to outliers.

Interpreting the RSF model with SHAP values has unveiled a dose–response relationship for the risk of death that changes its shape depending on dose range, is enhanced by fractionation/protraction of exposure, and is weakened by older age at the time of radiation exposure. Compared to prior studies, our application of these advanced analytic methods underscores the unique contributions of our study in the realm of radiation research. These novel findings not only extend our comprehension of the biological effects of high radiation but also pave the way for developing more robust radiation protection measures, especially in contexts where radiation exposure is a significant concern, such as space travel.

### Supplementary Information

Supplementary Information.

## Data Availability

The datasets generated during and/or analyzed during the study are available from the corresponding author upon request. Data used in this study were sourced from the Janus Archive for Mouse Aging Studies at Argonne National Laboratory.
