# Supplementary material for: Quantifying the effects of neutron dose, dose protraction, age and sex on mouse survival using parametric regression and machine learning on a 21,000-mouse data set
Source: Sci Rep. 2023 Dec 9;13:21841. doi: 10.1038/s41598-023-49262-3 (PMC10710496; doi:10.1038/s41598-023-49262-3)
Supplement: Supplementary file 1 — Supplementary Information. [file 41598_2023_49262_MOESM1_ESM.docx]

**Supplementary Material**

Supplementary Table 1. Summary statistics on some relevant variables in the analyzed data set.

| **Experi-ment** | **mean Sex (0=F, 1=M)** | **min Age at Death (days)** | **max Age at Death (days)** | **mean Age at Death (days)** | **min Treat-ment Age (days)** | **max Treat-ment Age (days)** | **max Dose (cGy)** | **mean Dose (cGy)** | **max Num-ber of Frac-tions** | **max Dura-tion of Frac-tion (min)** | **Total mice** |
| --- | --- | --- | --- | --- | --- | --- | --- | --- | --- | --- | --- |
| 2 | 0.54 | 113 | 1471 | 817.76 | 100 | 293 | 226.08 | 104.97 | 72 | 360 | 5207 |
| 3 | 0.68 | 156 | 1473 | 890.87 | 103 | 123 | 226.08 | 58.21 | 1 | 480 | 1669 |
| 4 | 0.55 | 206 | 1497 | 881.25 | 93 | 178 | 301.44 | 57.80 | 24 | 45 | 3731 |
| 7 | 0.61 | 204 | 1452 | 892.86 | 99 | 519 | 150.72 | 79.01 | 60 | 45 | 1528 |
| 8 | 0.64 | 145 | 1398 | 851.73 | 101 | 115 | 323.79 | 96.81 | 180 | 45 | 647 |
| 9 | 0.10 | 131 | 1517 | 967.91 | 101 | 123 | 37.68 | 5.17 | 24 | 45 | 3979 |
| 12 | 1.00 | 219 | 1461 | 799.14 | 116 | 116 | 226.08 | 178.93 | 6 | 20 | 537 |
| 13 | 0.51 | 143 | 1505 | 960.07 | 106 | 118 | 40.04 | 9.38 | 60 | 20 | 4010 |

**Supplementary Figure 1.** Cox Model R Output.

1. Model with main effects only.


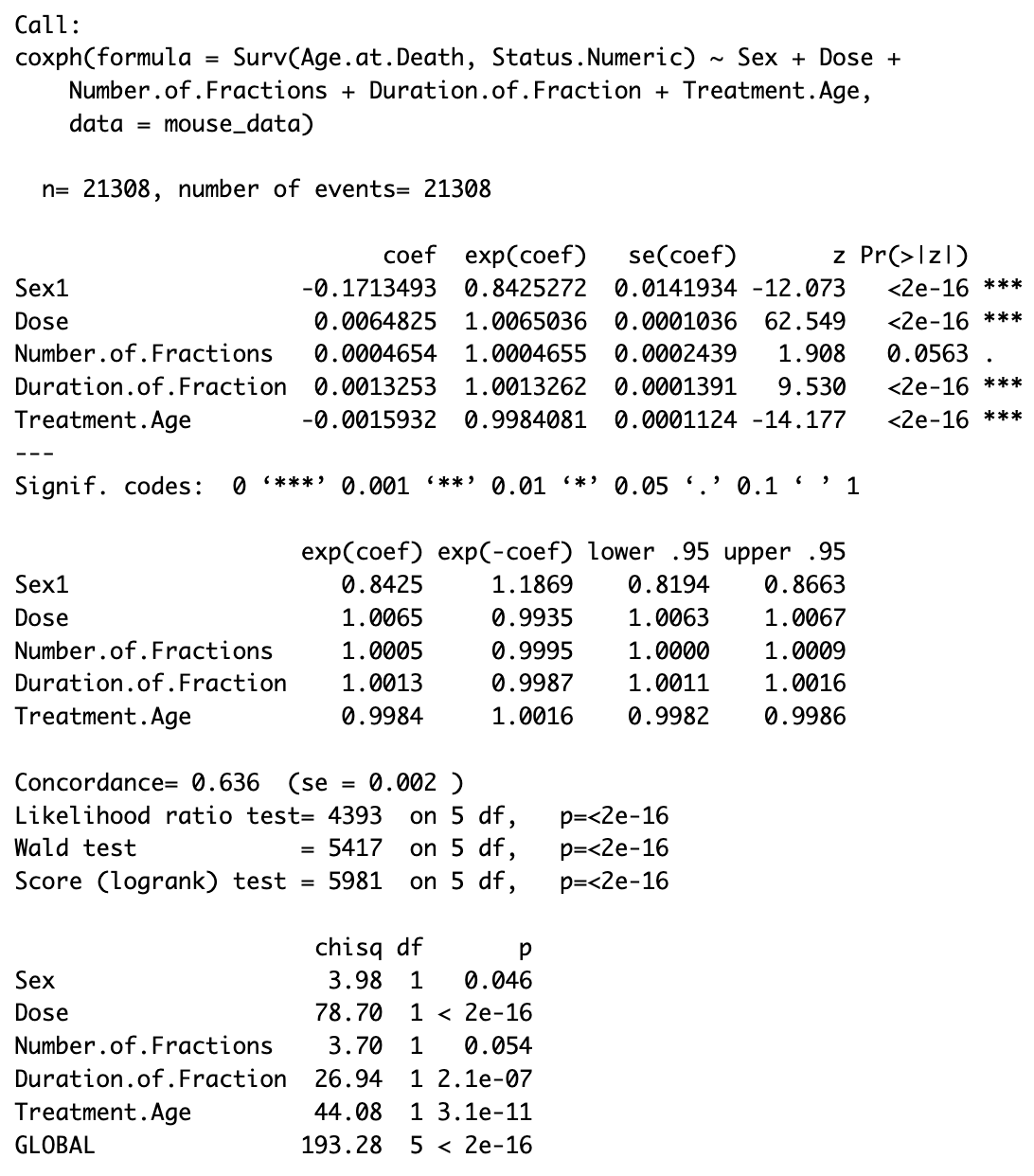


1. Model with main effects and interaction terms.


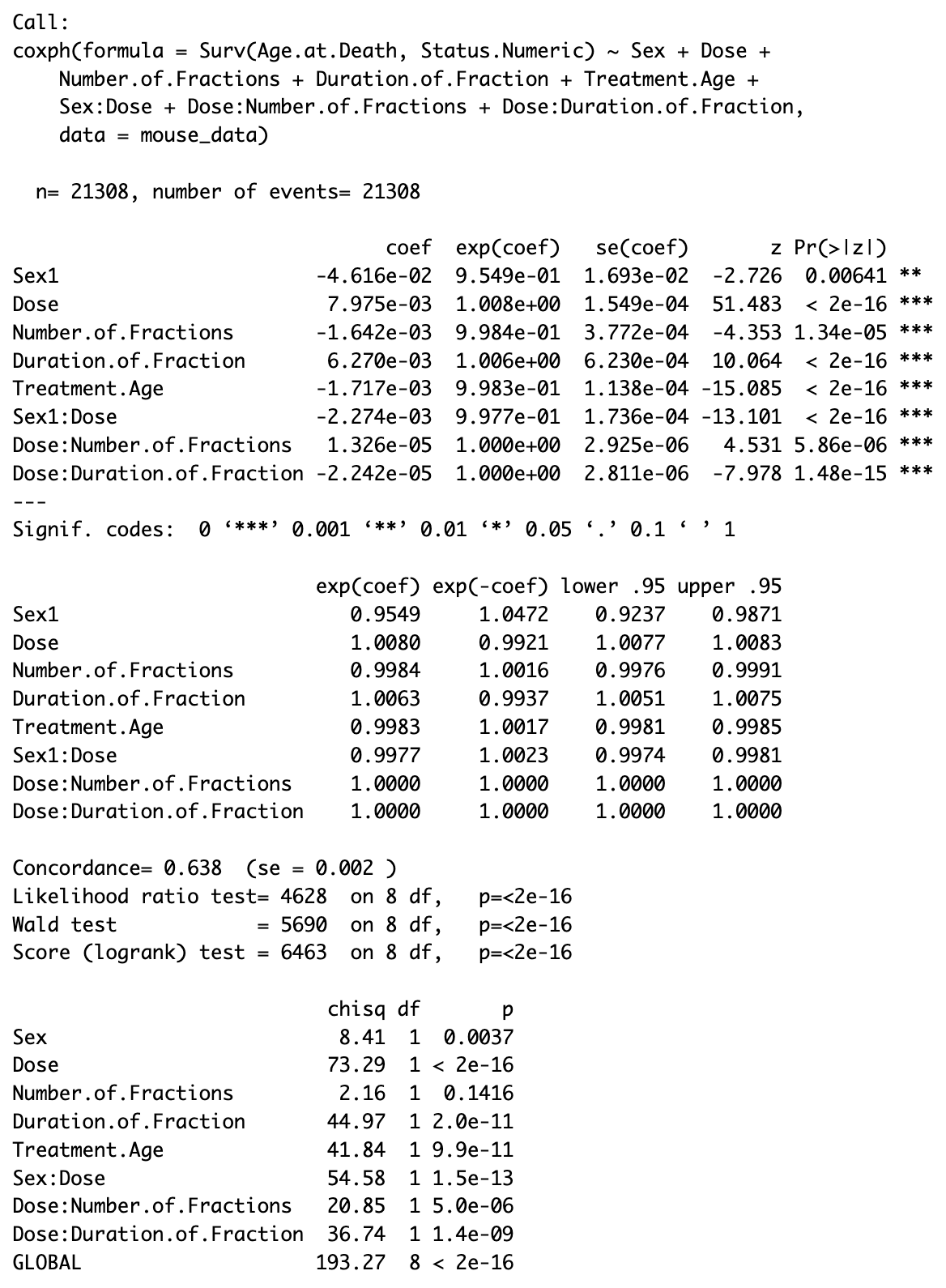


**Supplementary Figure 2.** Martingale Residuals for the fitted Cox model as function of Duration of Fraction.
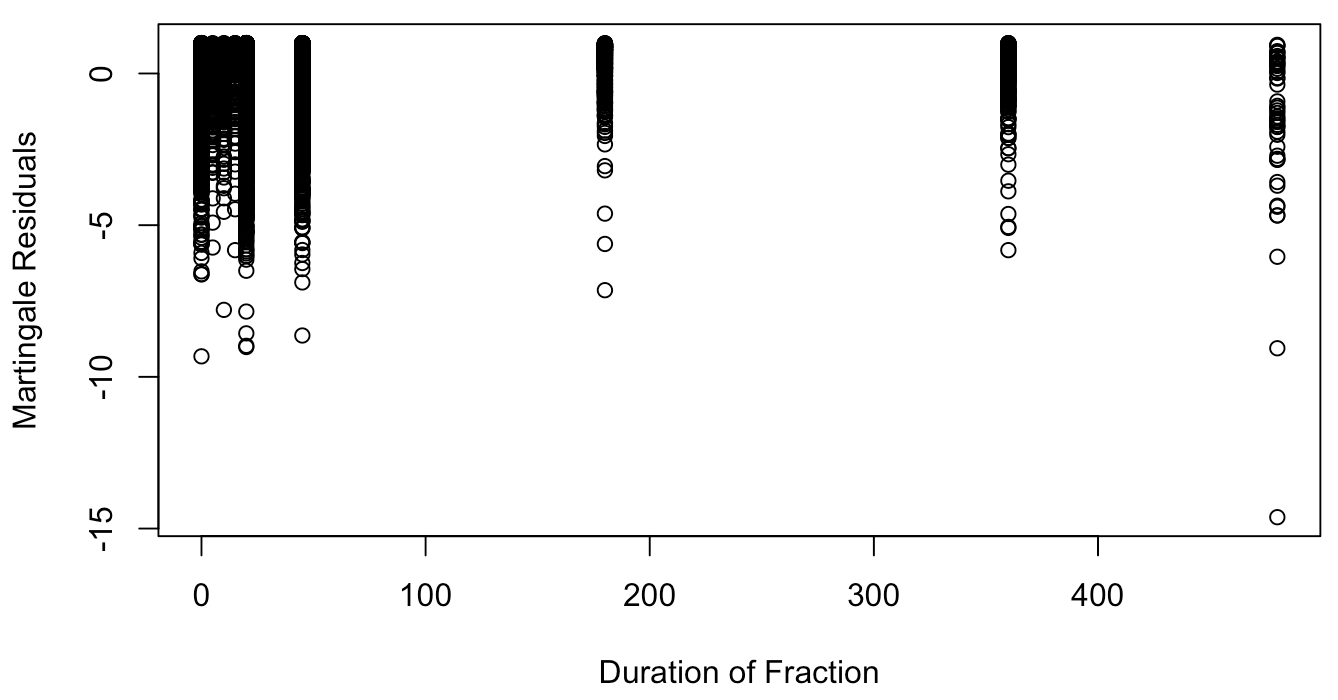


**Supplementary Figure 3.** Martingale Residuals for the fitted Cox model as function of Number of Fractions.
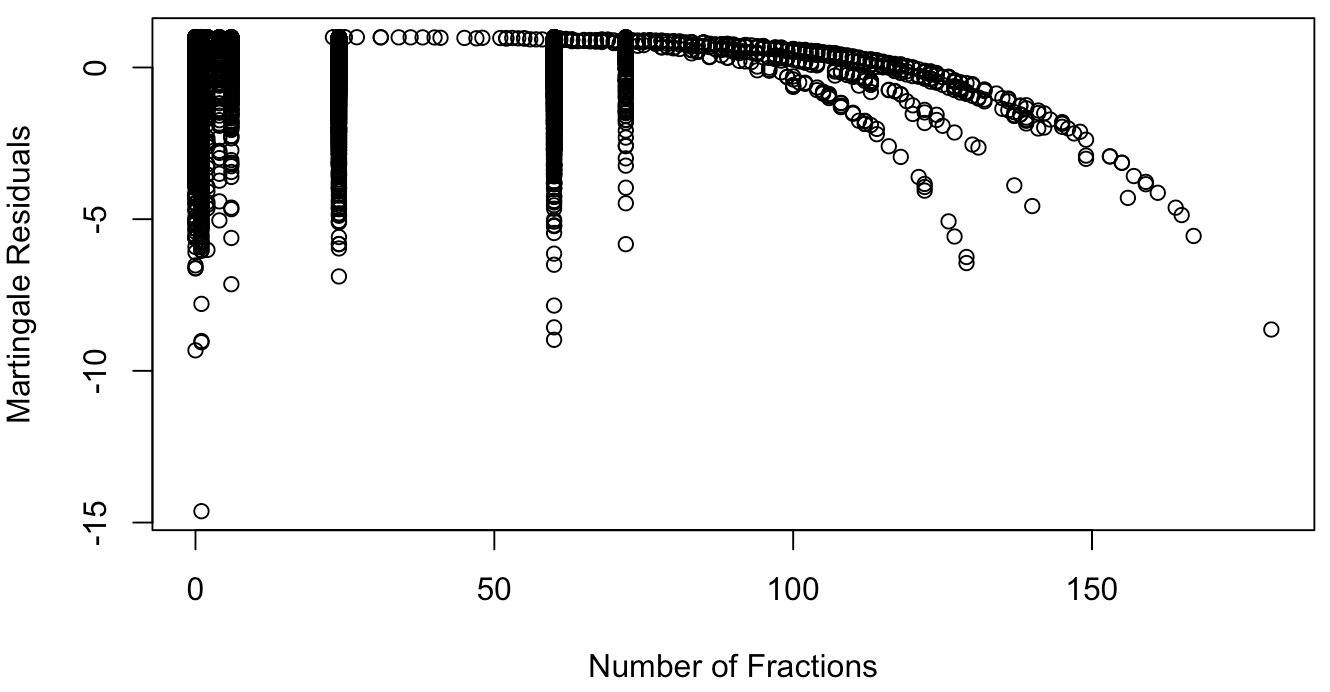


**Supplementary Figure 4.** Martingale Residuals for the fitted Cox model as function of Dose.
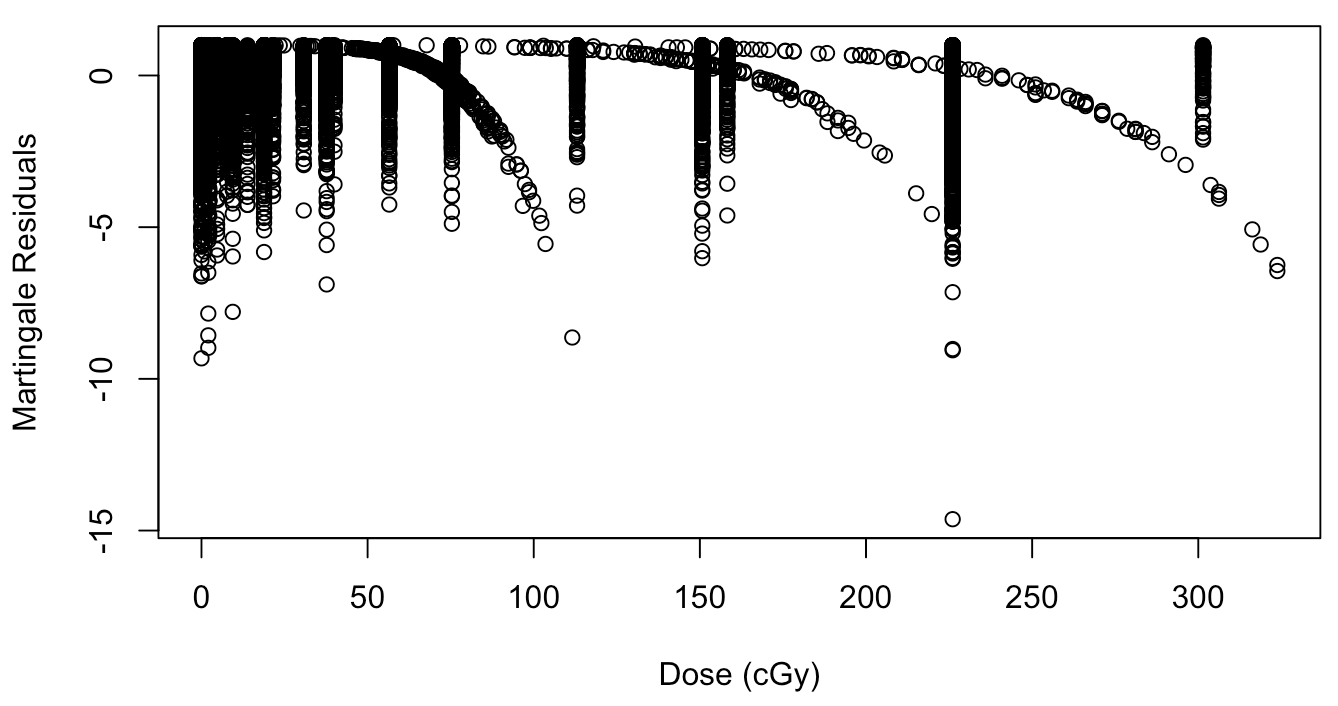


**Supplementary Figure 5.** Martingale Residuals for the fitted Cox model as function of Treatment Age.
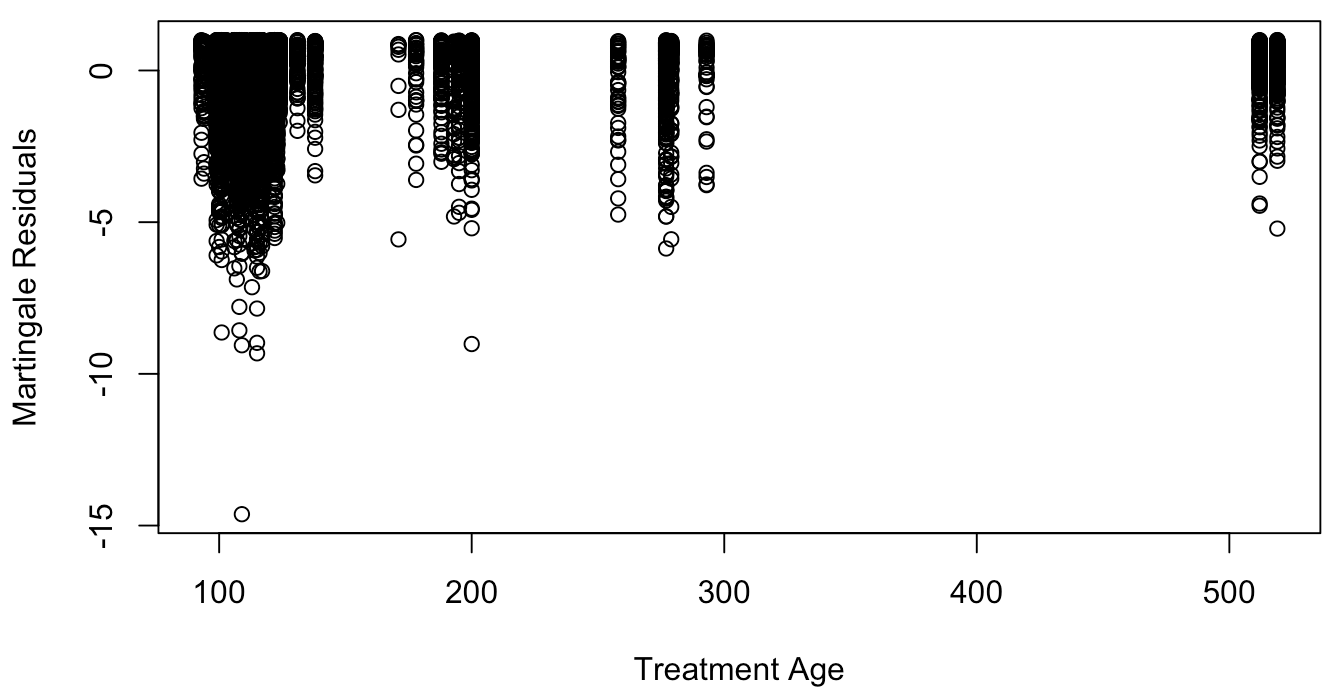


**Supplementary Figure 6.** Plot visualizing the scaled Schoenfeld residuals for Time versus Beta(t) for Sex.
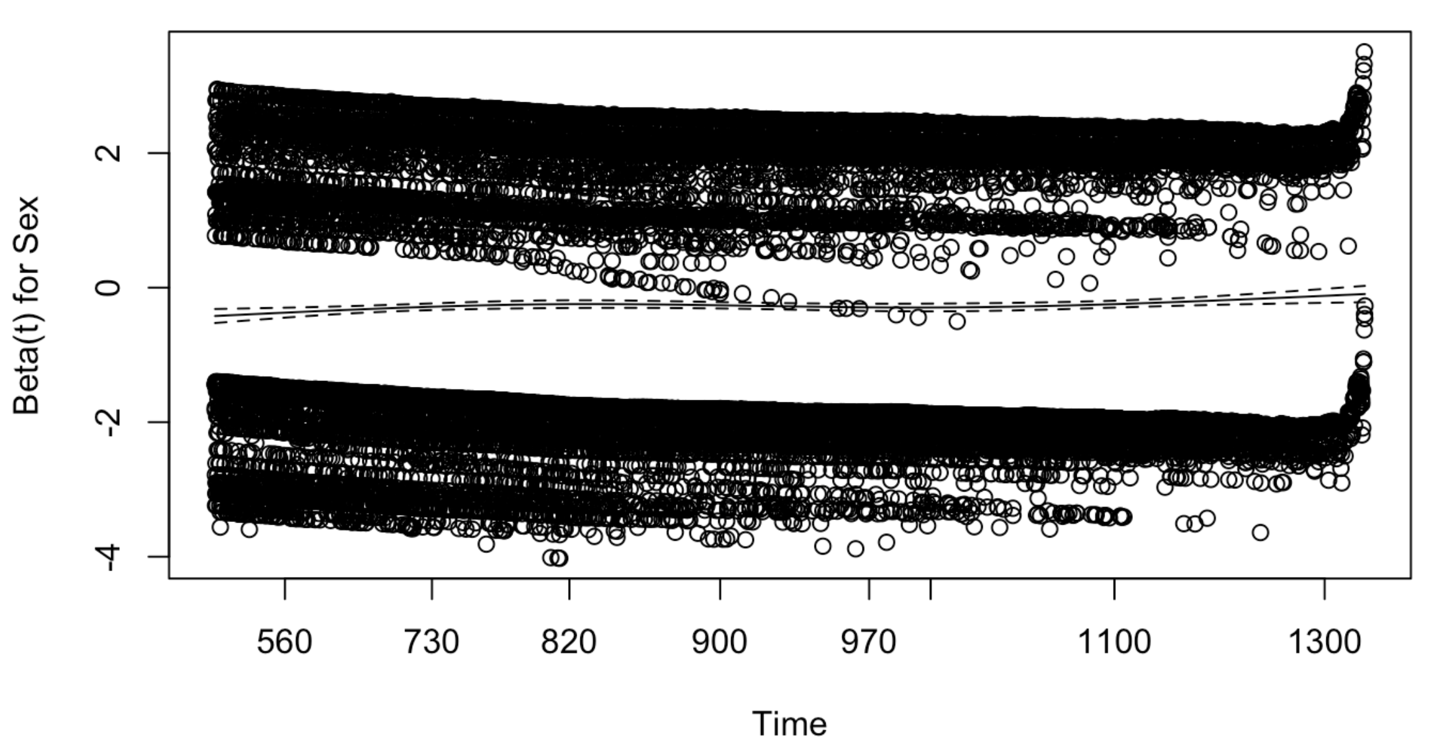


**Supplementary Figure 7.** Plot visualizing the scaled Schoenfeld residuals for Time versus Beta(t) for Treatment Age.
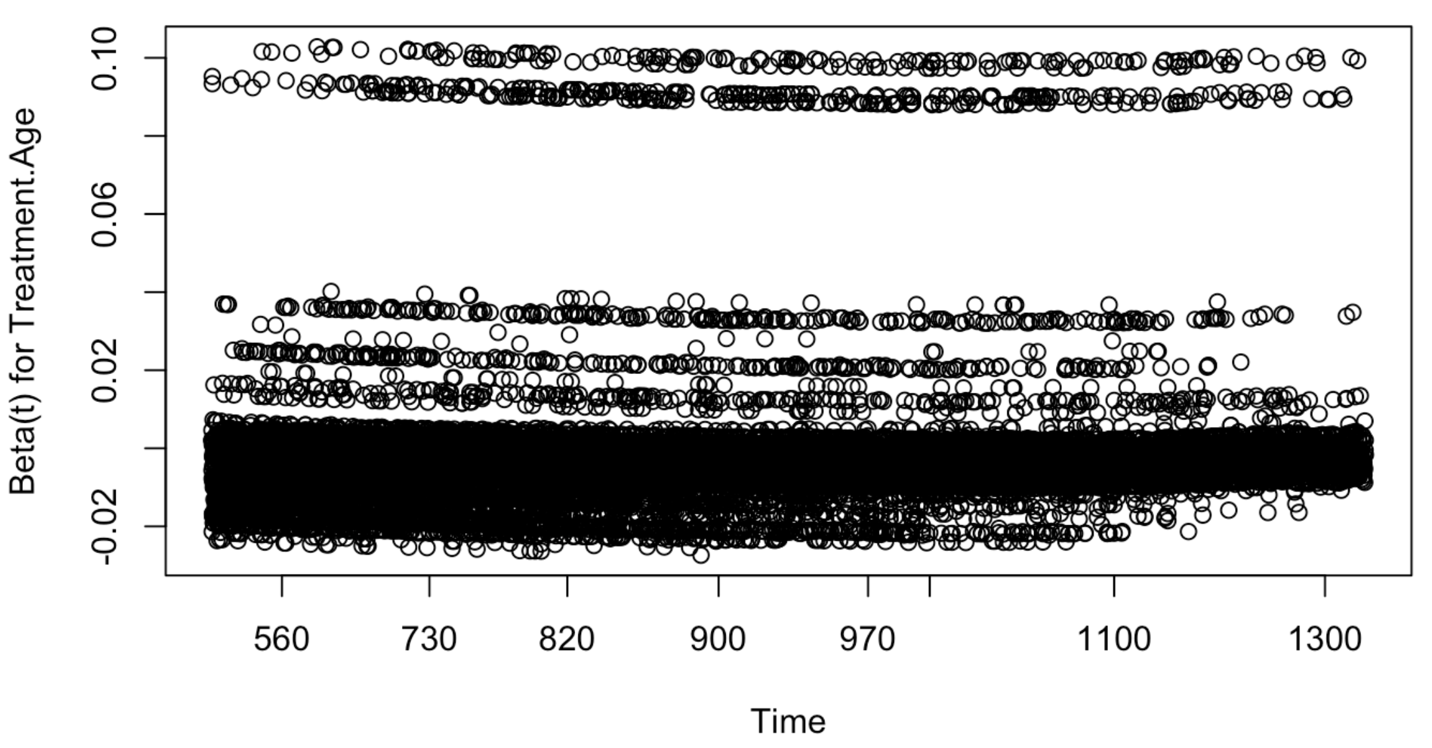


**Supplementary Figure 8.** Plot visualizing the scaled Schoenfeld residuals for Time versus Beta(t) for Dose.
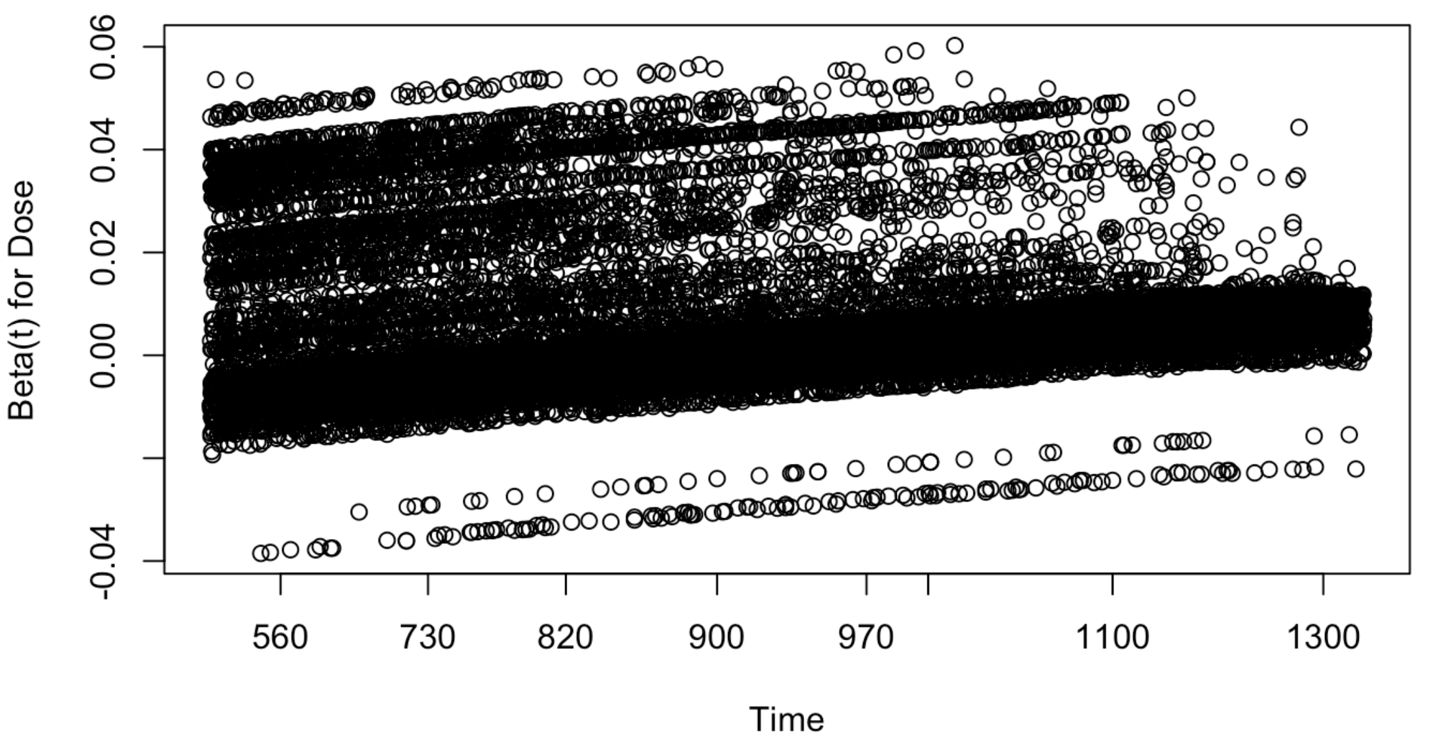


**Supplementary Figure 9.** Plot visualizing the scaled Schoenfeld residuals for Time versus Beta(t) for Number of Fractions.
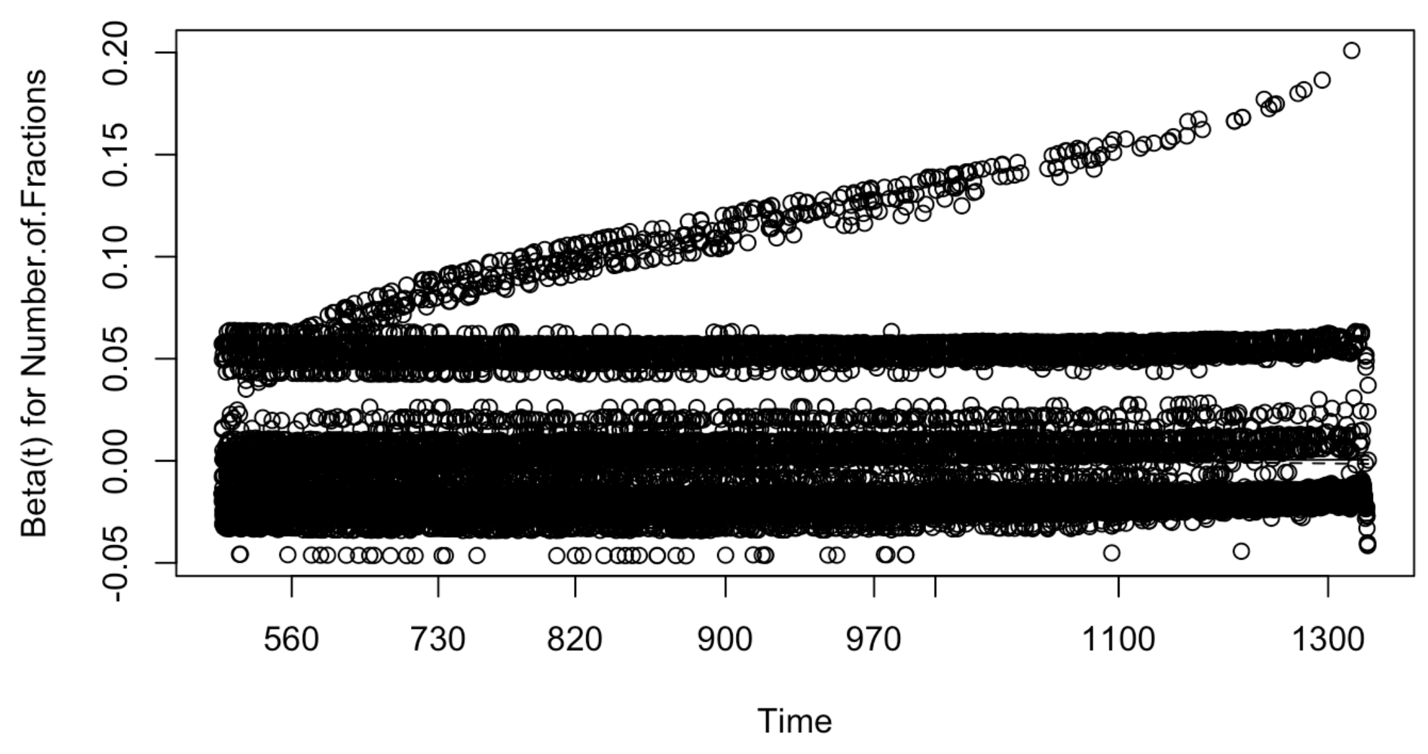


**Supplementary Figure 10.** Plot visualizing the scaled Schoenfeld residuals for Time versus Beta(t) for Sex.


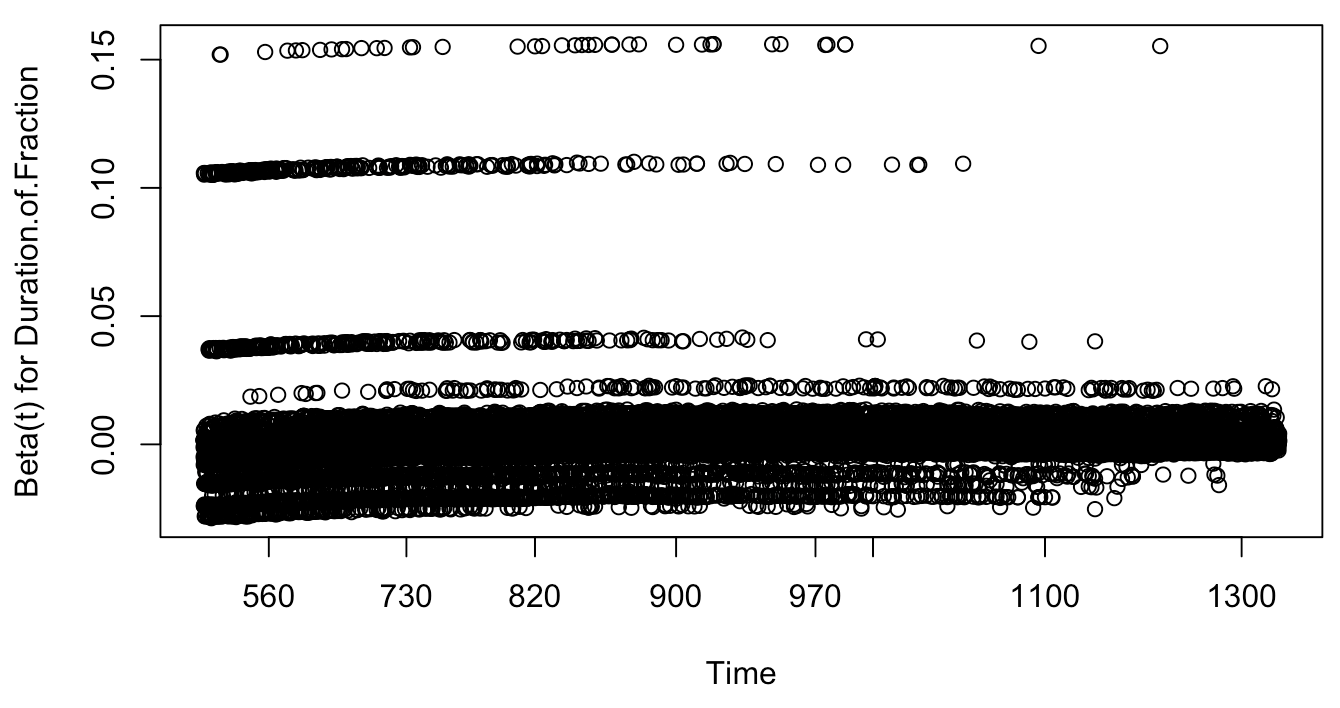


**Supplementary Figure 11.** Distributions of concordance (c-index) values generated by the Cox model on training versus testing data sets.

**
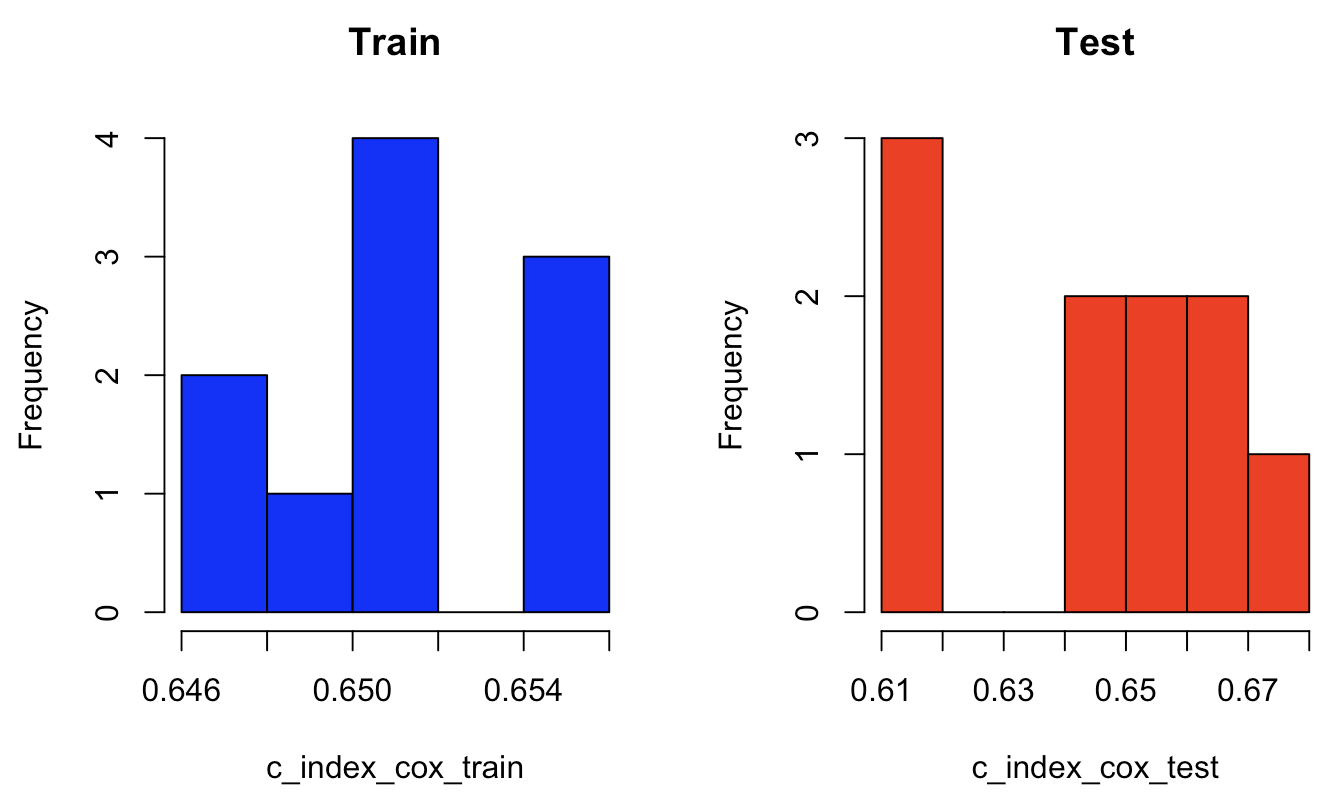
**

**Supplementary Figure 12.** Pearson correlation matrix on the training data set (randomly selected 70% of the data).

**
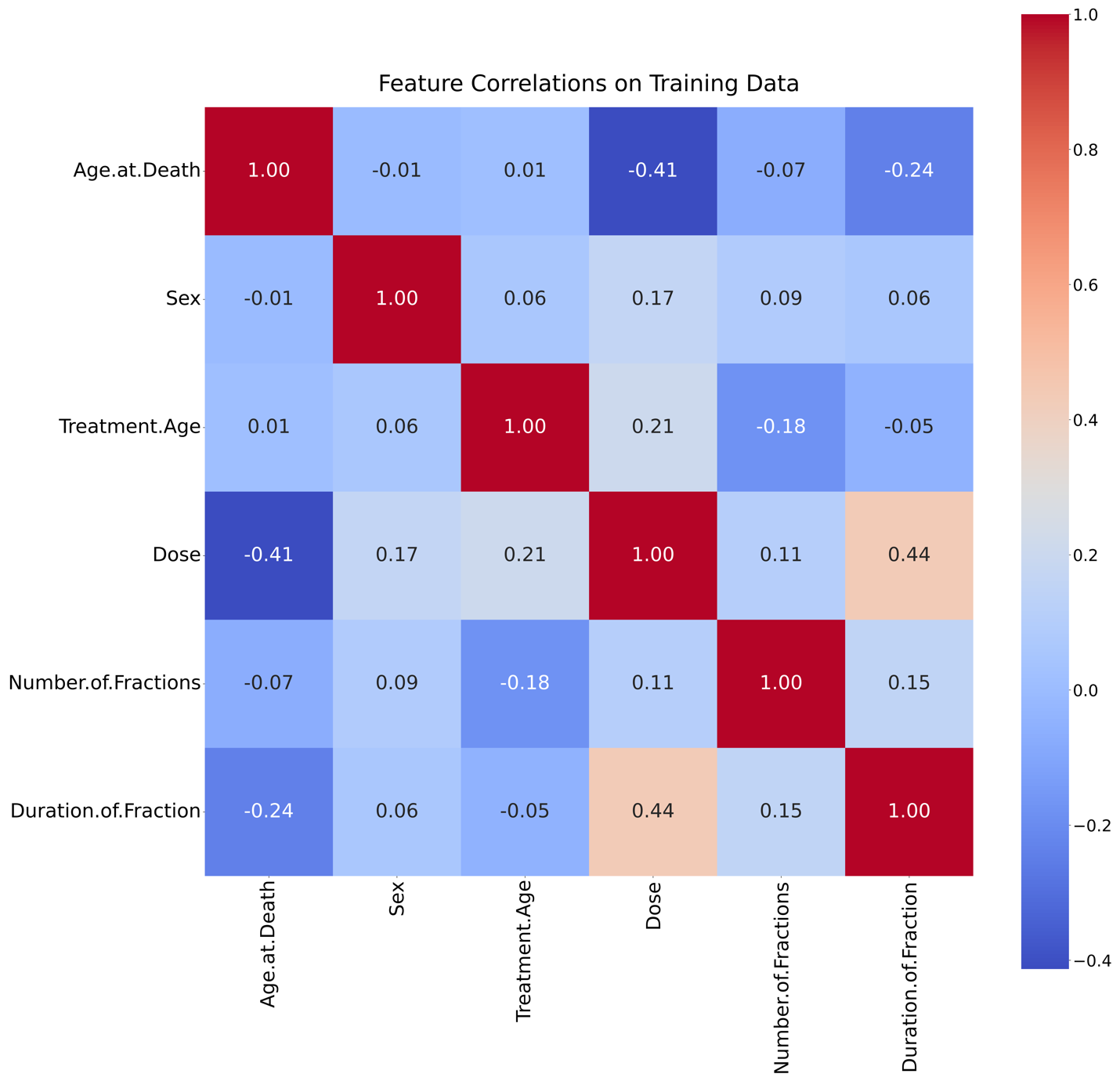
**

**Supplementary Figure 13.** Correlations between Dose, Treatment Age, and Sex, and their SHAP Values.


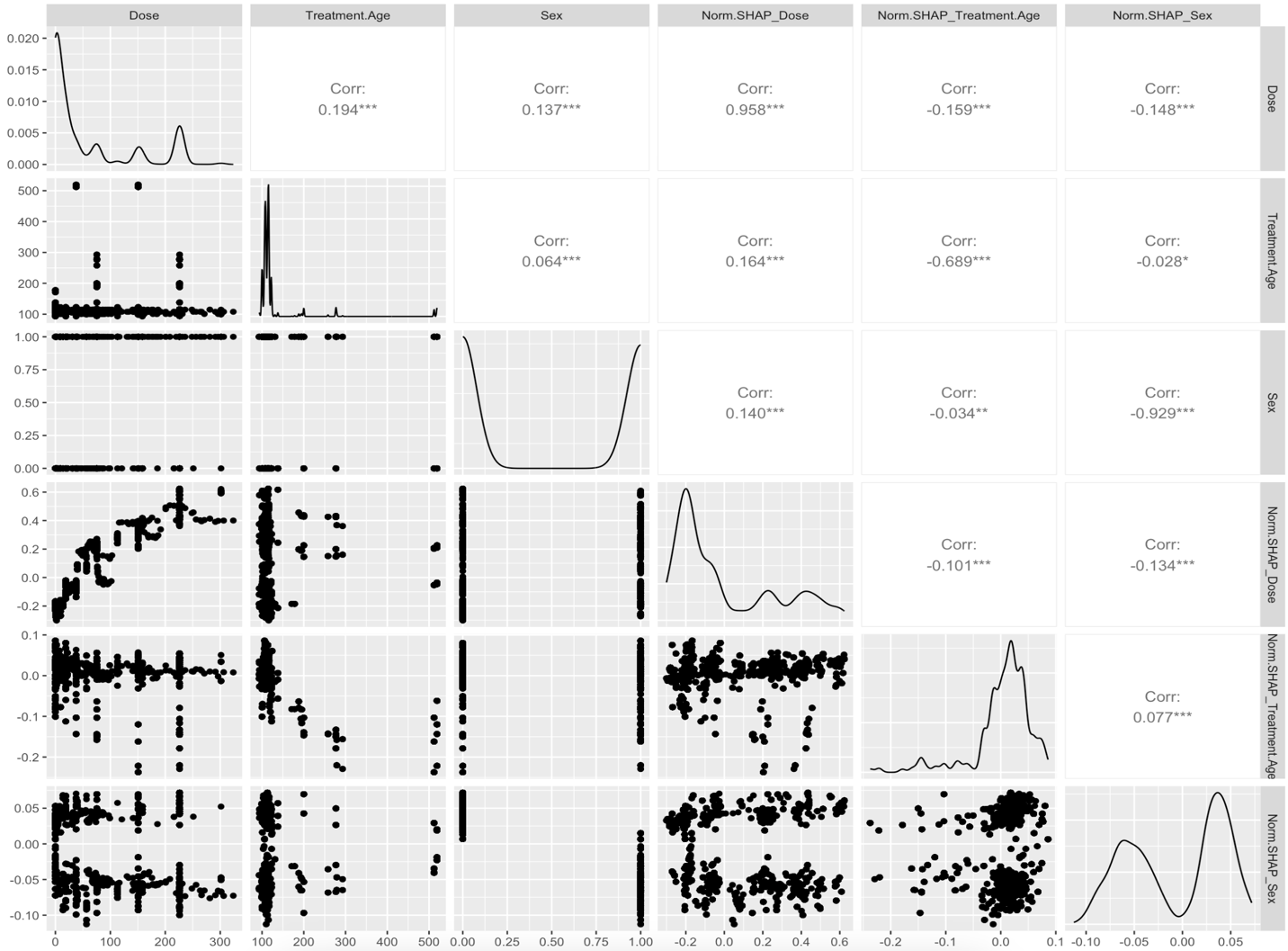


**Supplementary Figure 14.** Correlations between Dose, Number of Fractions, Duration of Fraction, and their SHAP Values.


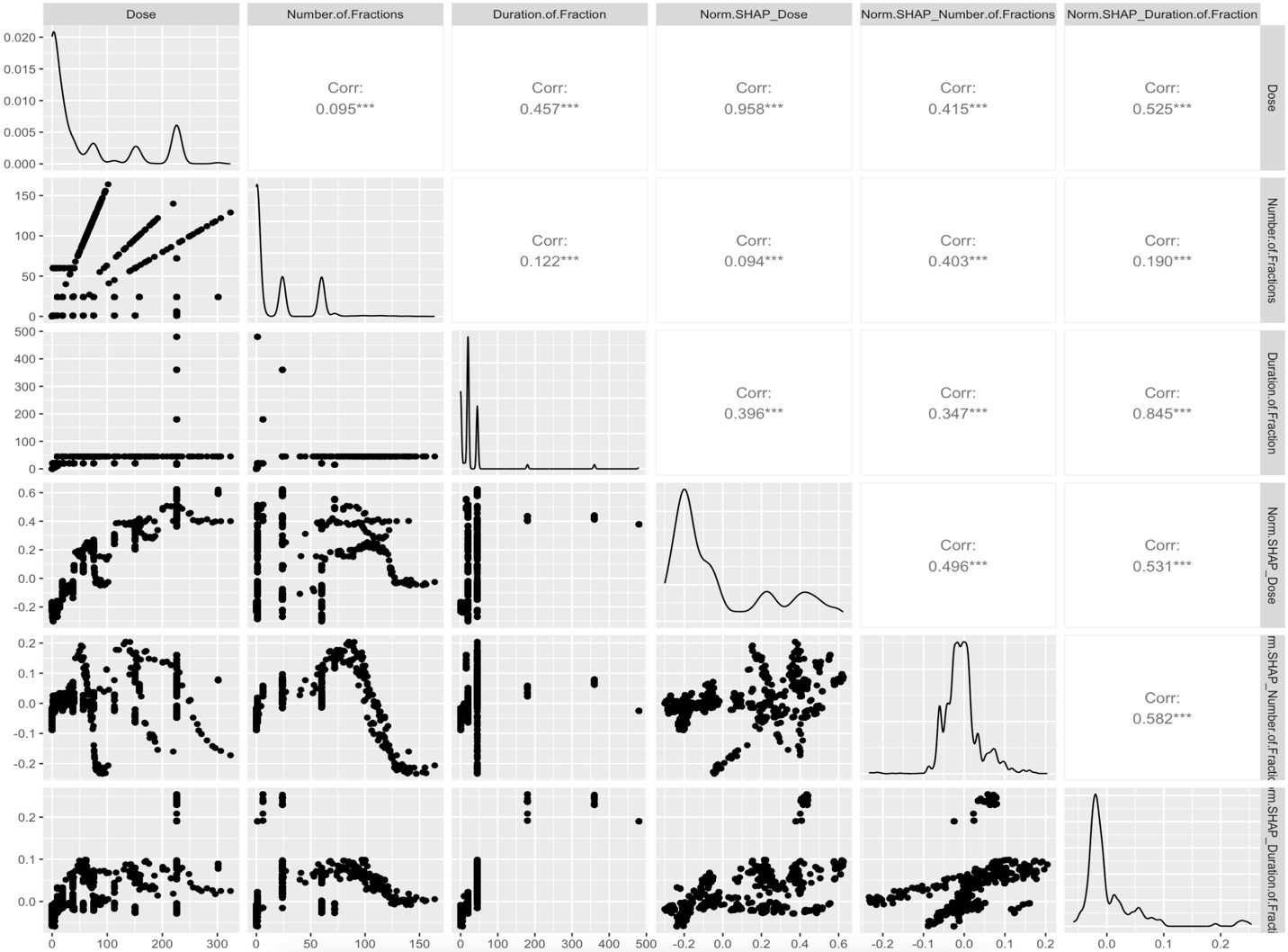


**Supplementary Figure 15.** Correlations between all variables of interest and their SHAP Values.

**
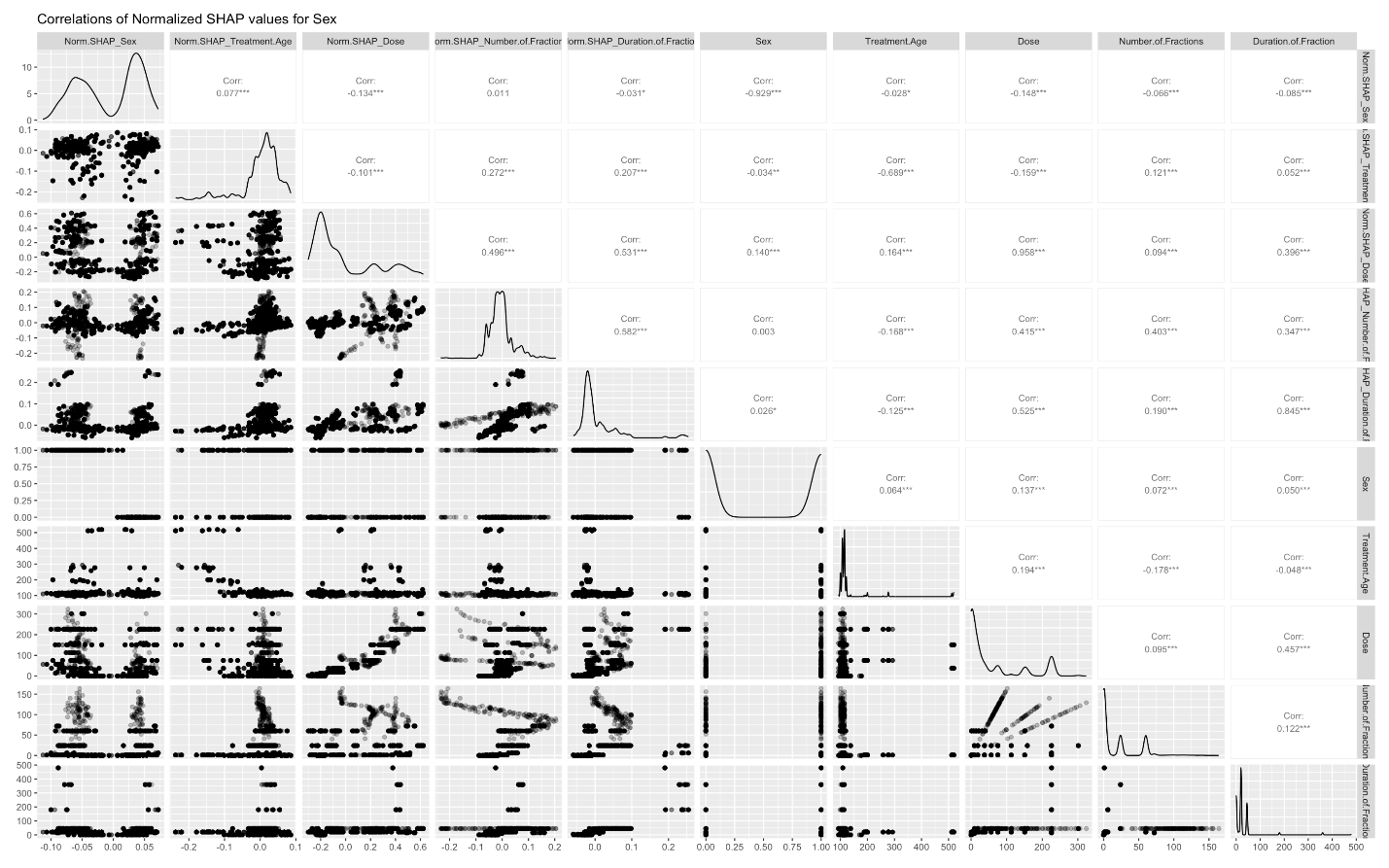
**
